# Conservation publications and their provisions to protect research participants

**DOI:** 10.1111/cobi.13337

**Published:** 2019-06-14

**Authors:** Harriet Ibbett, Stephanie Brittain

**Affiliations:** ^1^ Department of Zoology University of Oxford 11a Mansfield Road Oxford OX1 3SZ U.K.

**Keywords:** anonymity, human research ethics, hunting, informed consent, institutional review boards, interviews, rule breaking, social science, anonimato, cacería, ciencias sociales, comités de revisión institucional, consentimiento autorizado, entrevistas, ética de la investigación humana, rompimiento de reglas

## Abstract

Social science methods are increasingly applied in conservation research. However, the conservation sector has received criticism for inadequate ethical rigor when research involves people, particularly when investigating socially sensitive or illegal behaviors. We conducted a systematic review to investigate conservation journals’ ethical policies when research involves human participants, and to assess the types of ethical safeguards documented in conservation articles. We restricted our review to articles that used social science methods to gather data from local people about a potentially sensitive behavior: hunting. Searches were conducted in the Web of Science, Scopus, and Google Scholar for research articles in English published from January 2000 to May 2018. Only studies conducted in countries in south and Southeast Asia, Africa, and Central and South America were considered. In total, 4456 titles and 626 abstracts were scanned, with 185 studies published in 57 journals accepted for full review. For each article, any information regarding ethical safeguards implemented to protect human participants was extracted. We identified an upward trend in the documentation of provisions to protect human participants. Overall, 55% of articles documented at least one ethical safeguard. However, often safeguards were poorly described. In total, 37% of journals provided ethics guidelines and required authors to report ethical safeguards in manuscripts, but a significant mismatch between journal policies and publication practice was identified. Nearly, half the articles published in journals that should have included ethics information did not. We encourage authors to rigorously report ethical safeguards in publications and urge journal editors to make ethics statements mandatory, to provide explicit guidelines to authors that outline journal ethical reporting standards, and to ensure compliance throughout the peer‐review process.

## Abstract (es)

Publicaciones sobre Conservación y sus Aportaciones para Proteger a los Participantes de la Investigación

**Resumen**

La metodología de las ciencias sociales cada vez se aplica más en la investigación para la conservación. Sin embargo, el sector de la conservación ha recibido críticas por un rigor ético inadecuado cuando la investigación involucra a las personas, particularmente cuando se investigan comportamientos ilegales o socialmente sensibles. Realizamos una revisión sistemática para investigar las políticas éticas de las revistas de conservación cuando la investigación involucra a participantes humanos y para evaluar los tipos de salvaguardas éticos documentados en los artículos de conservación. Restringimos nuestra revisión a artículos que usaron metodología social para recolectar datos de la gente local sobre un comportamiento potencialmente sensible: la cacería. Las búsquedas se realizaron en los sitios Web of Science, Scopus y Google Scholar enfocadas en los artículos de investigación en inglés publicados entre enero del 2000 y hasta mayo del 2018. Solamente consideramos los estudios realizados en los países del sur y sureste de Asia, África y Centro y Sudamérica. Un total de 4456 títulos y 626 resúmenes fueron escaneados. Revisamos completamente 185 estudios publicados en 57 revistas aceptadas. Se extrajo cualquier información con respecto a los seguros éticos implementados para proteger a los participantes humanos. En general, el 55% de los artículos documentaron al menos un seguro ético. Sin embargo, con frecuencia los seguros eran descritos pobremente. En total, el 37% de las revistas proporcionaron guías éticas y requirieron que los autores reportaran los seguros éticos en los manuscritos, pero se identificó una discrepancia significativa entre las políticas de las revistas y las prácticas de publicación. Casi la mitad de los artículos publicados en las revistas que deberían haber incluido información ética no la tuvo. Alentamos a los autores a que reporten con rigurosidad los seguros éticos en las publicaciones y les urgimos a los editores de dichas revistas que hagan obligatorias las declaraciones éticas, que proporcionen a los autores guías explícitas que resuman los estándares de reportaje ético de las revistas en línea, y que aseguren el cumplimiento por medio del proceso de revisión por pares.

*Article impact statement*: Conservation articles often poorly describe ethical safeguards, and journals should ensure authors report ethical considerations.

## Introduction

There has been a push to adopt social science methods in conservation (Büscher & Wolmer 2007; Sutherland et al. 2018) in recognition of the ability of social science research to improve conservation practice (Bennett et al. 2017) and the realization that understanding social systems is imperative to achieve conservation objectives (Mascia et al. 2003; Milner‐Gulland 2012). Yet, despite the increasing emphasis on interdisciplinarity, social scientists remain a minority within the discipline (Bennett et al. 2016). Typically, conservation scientists are trained in natural sciences (Fox et al. 2006) and are often poorly equipped to undertake research that requires extensive knowledge of social science techniques (Campbell 2005; Drury et al. 2011). Thus, conservation scientists have been criticized for poor social science research practice and for applying insufficient ethical rigor when conducting research that involves human participants (St. John et al. 2014). Conservationists have been accused of thoughtless disclosure of research findings and failure to consider the ethical implications of research (Solomon et al. 2016; St. John et al. 2016).

Ethics underpins conservation partly because the founding principles of the discipline stem from ethical beliefs in the intrinsic value of biodiversity, but also because the science of conservation is ethically contentious (Van Houtan 2006; Miller et al. 2011; Robinson 2011). A primary aim of conservation science is to enact positive change for the benefit of biodiversity (Meine et al. 2006; Kareiva & Marvier 2012), yet doing so often negatively affects the well‐being of communities who depend on that biodiversity (Agrawal & Redford 2009; McKinnon et al. 2016). Moreover, conservation research is frequently conducted where people are poor and dependent on natural resources; in cultural contexts where gender roles differ and power imbalances prevail; and against backdrops where conservation is a concept imposed by outsiders (Dowie 2009). If these factors are inadequately considered, research risks infringing on human rights (Brockington et al. 2006).

Researchers have moral, professional, and increasingly legal obligations to act ethically and with good moral intent (Wiles 2013). Research ethics build on the principles of beneficence, whereby the dignity, rights, autonomy, and welfare of participants are protected; nonmaleficence, whereby it is ensured research does not cause harm; and justice, whereby the benefits and burdens of research are distributed equally among participants (Aluwihare‐Samaranayake 2012; Wiles 2013). Beyond simply being the right thing to do (Vanclay et al. 2013), implementing these ethical principles in research helps maintain the trust and social goodwill of the various publics with and for whom researchers work and assures research integrity (Israel & Hay 2006). This is particularly important because much research occurs unobserved, providing significant scope for improper conduct. By demonstrating ethical behavior, researchers promote confidence in the research process and in the reliability of research findings (Israel & Hay 2006).

Various guidelines and codes of conduct exist to inform ethical research practice when working with people. Typically, these provide frameworks for thinking through ethical dilemmas when they arise (Wiles 2013) and are produced by governments, funders (e.g., U.K. Economic and Social Research Council [ESRC]), and professional associations of specific disciplines. For example, the Codes of Ethics of the American Association of Anthropology (2019) and British Psychological Society (2016) outline discipline‐specific ethical guidelines that elaborate and extend national and international guidelines. Within conservation, the Code of Ethics of the Society for Conservation Biology (SCB) acknowledges that research should always “Protect the rights and welfare of human subjects used in research” (SCB 2004). Conservationists often referred to ethical codes established for sociology, geography, and anthropology for guidance (Watson 2010).

Over the last 2 decades, the regulation of ethics in research has increased substantially (Wiles 2013); most western institutions now require researchers to obtain ethical approval from institutional review boards (IRBs) (also known as ethical review boards, research ethics boards, and research ethics committees) prior to commencing research (Dyer & Demeritt 2009; Speiglman & Spear 2013). This process is underpinned by the concept of informed consent, whereby the purpose, methods, intended uses of research are described (Wax 1980; Guillemin & Gillam 2004) and the risks and benefits of participation are discussed in a language understandable to participants before research commences (Speiglman & Spear 2013; ESRC 2015). Informed consent, if sought diligently, should ensure that potential participants are not coerced and are provided with all the information they need to make informed and independent decisions about whether to participate. Where appropriate, IRBs may also require researchers to assure anonymity (participants remain unidentified) or confidentiality (researchers protect information from being discovered by others) or both (Vanclay et al. 2013; Wiles 2013), particularly when researching socially sensitive or illegal behaviors, such as hunting.

Today, overhunting poses one of the greatest threats to wildlife worldwide and is a high priority for conservation research (Ripple et al. 2016; Benitez‐Lopez et al. 2017). Social science methods, such as interviews, have risen in prominence in conservation research (Newing 2010; Young et al. 2018) and are increasingly applied in hunting research to profile those involved (e.g., Harrison et al. 2015); quantify the prevalence of damaging behaviors (e.g., Nuno et al. 2013); and identify why these behaviors occur (e.g., Knapp 2012). Findings may result in measures to protect biodiversity by, for example, increasing the enforcement of environmental rules (St. John et al. 2016).

However, investigating hunting presents specific ethical challenges. In many contexts hunting wildlife is illegal; thus, research requires respondents to incriminate themselves or their community by reporting information about rule breaking (Solomon et al. 2007). Although results may be critical for conservation outcomes, the ethics of placing people in such positions is questionable. Participants may hesitate to engage in research and be wary of reporting the truth due to potential repercussions; resultant data may be biased (Gavin et al. 2010). Although measures can be adopted to assure greater anonymity and militate against dishonest or biased responses (Nuno & St. John 2015), research endows researchers with influential knowledge, which if published thoughtlessly may disproportionately affect all stakeholders involved (St. John et al. 2016).

During the publication process, peer review provides a critical point at which the quality, scientific excellence, and ethical integrity of research is assessed and endorsed (Solomon 2007). Once published, articles become reference points for the development of future research and ethical practice. Yet, when reading articles to develop the design of our own research, we observed that human research ethics were rarely addressed or mentioned in published literature, a trend also noted by Young et al. (2018) in a review of the use of interviews in conservation research. This is concerning as publishing articles without the inclusion of ethical safeguards risks validating low‐quality social science and perpetuates poor research practice (St. John et al. 2014).

In response, we undertook a systematic review to assess the extent to which ethical safeguards are described in peer‐reviewed conservation publications. We recognize research ethics is a broad field that embodies many issues. We focused only on measures to protect human participants documented by authors in articles. We specifically selected research for which social science methods were used to investigate wildlife hunting as a case study because this reflects our own area of expertise and because of the specific ethical challenges associated with conducting research on rule breaking. Our specific research objectives were to review the guidelines journals offer to authors, and the ethical standards they require authors to report on to infer, based on information about ethical safeguards included in articles, the extent to which conservation studies adhere to currently accepted ethical standards for research practice that involves human participants; and to describe the types of ethical safeguards documented by authors to protect human research participants.

## Methods

### Search Criteria and Selection

Between August 2017 and May 2018, we searched Google Scholar, Scopus, and the Web of Science with the search term “*hunting* OR *wildlife* OR *hunter* OR *bushmeat* OR *wild meat* OR *poaching* OR *poacher* AND *interview*.” We selected this phrase because it encompasses the broad ways in which hunting and those who hunt are described in the literature. *Interview* is a common umbrella term used to describe collecting data from people. For example, people are interviewed when questionnaires or surveys are administered, and interviews frequently precede or compliment other modes of data collection.

We restricted searches to English language research articles published from January 2000 to May 2018. We confined our search through Web of Science and Scopus to articles published in conservation biology, ecology, and zoology journals. Although this excluded relevant research published in interdisciplinary journals, we specifically aimed to assess the levels of human ethics reporting by conservation scientists, who we assumed usually target peer‐reviewed conservation journals for publication. Google Scholar did not permit restrictions by journal subject; therefore, some articles published in journals with wider scopes were also included. Searches produced 4456 studies. For full review guidelines, see Supporting Information.

Each title was scanned independently by H.I. and S.B. Cohen's Kappa Statistic was used to assess the level of agreement between examiners when accepting or rejecting titles. A result of 0.67 suggested substantive agreement (Watson & Petrie 2010). In total, 626 articles were accepted for abstract review.

When reviewing abstracts, we accepted articles that referenced the use of interviews or any other social science method that involved gathering data from local people (e.g., self‐reporting, questionnaire, or focus‐groups) to investigate hunting of terrestrial species. We restricted our search to studies that investigated the hunting activities of local communities in countries situated in south and Southeast Asia, Africa, and Central and South America because this is where most research effort is concentrated and ethical issues arise. Studies that featured trophy or sports hunting were excluded. Overall, 185 studies were accepted for full review.

### Data Extraction

Articles were randomly allocated between authors, and information was extracted on journal name, research country, species of research interest, legal status of hunting, research methods, and the ethical safeguards for human participants documented. We assessed the rigor with which authors reported on ethical safeguards by identifying whether the following 4 criteria were reported on: authors acknowledged research underwent formal ethical review by an IRB or equivalent, or an IRB reference was given; authors acknowledged that participant consent was sought; authors acknowledged research participants or communities were offered assurances of anonymity or confidentiality or both; and authors acknowledged that research was developed or conducted following a recognized ethical code of conduct.

Although these narrow criteria excluded other ethical issues such as data protection and data security, they represent the basic ethical safeguards IRBs require researchers to consider during research (ESRC 2015). Although it could be assumed that if IRB approval was reported then safeguards such as consent, anonymity, and confidentiality were also likely to have been considered as part of the ethical review process, we aimed to test whether they were independently reported. In recognition that IRBs are not present in every institution and that some researchers may follow ethical codes of conduct instead of, or in addition to, IRBs, we also included this as a fourth assessment criterion. Each of us read and cross‐checked all articles. If uncertainty arose, we discussed articles on a case‐by‐case basis and the decision we agreed on was recorded.

### Journal Requirements

We gathered data on the ethics policy of each journal featured in the review. In June 2018, we visited the website of each journal and extracted all relevant information. Usually information was located in a guidelines‐to‐authors section. We coded results to identify the types of ethical safeguards journals required authors to confirm adherence to and report on in manuscripts. We contacted journal editors via email and asked each to complete a short questionnaire (Supporting Information) about when ethics policies were introduced, the composition of the editorial board (proportion of social vs. natural scientists), and the journals’ peer‐review process. A follow‐up email was sent 2 weeks after the first to prompt responses.

### Statement on Human Subjects

Prior to contacting journal editors, we sought ethical guidance from the University of Oxford Central University Research Ethics Committee. We were advised ethical approval was not required for this research. Nonetheless, we sought free, prior, and informed consent from all journal editors and assured individuals that their responses would remain anonymous and confidential.

## Results

### Sample Characteristics

A total of 185 articles were published in 57 different academic journals. As expected, the majority of articles were published in journals dedicated to conservation, ecology, or zoology (*n* = 178, 96% [Supporting Information]). The sample also included 7 articles published in social science or generic science journals such as *PLoS One*. In total, 53% of articles were published in 5 journals (Supporting Information). Seventy‐six percent of reviewed articles were published after 2010, suggesting both increased research interest and increased adoption of social survey techniques to investigate hunting.

**Figure 1:**
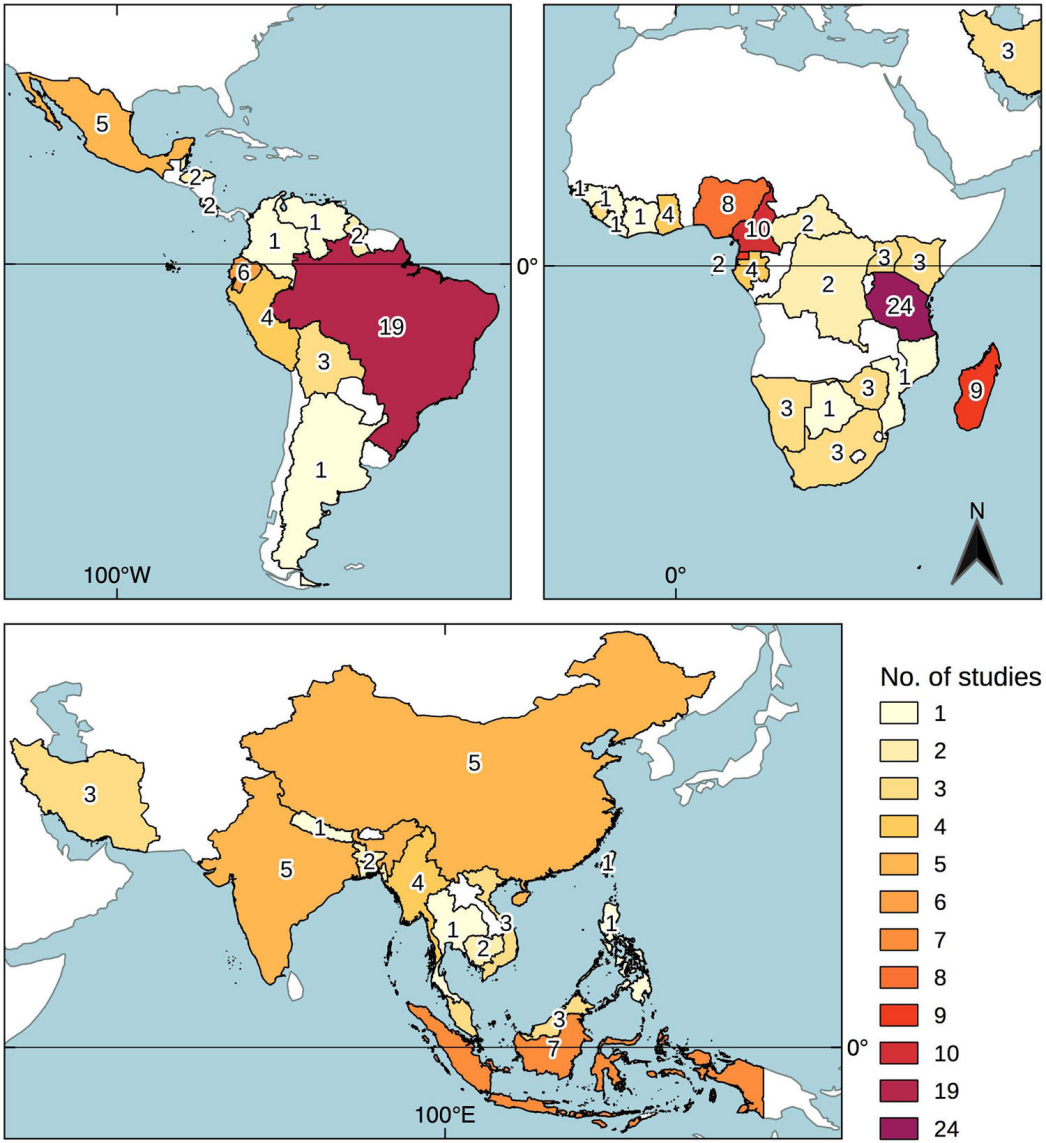
Location of studies featured in systematic review that used social research methods to collect information from local people about hunting behavior.

Fifty‐two percent of studies were undertaken in Africa, 26% in Latin America, and 22% on the Asian subcontinent (Fig. 1 & Supporting Information). There was a bias in research effort with over one‐fifth of studies conducted in one of 2 countries: Tanzania or Brazil. Articles were published by 153 different authors; 24 authors accounted for 31% of the publications (Supporting Information). Only 29% of articles were first authored by a researcher based at an institution in the country where research was conducted. We found several authors published multiple articles from a specific site or country, presumably from research conducted as part of an extended study. These were considered separate data points, our justification being that although authors may have used the same ethical guidelines throughout their research, their reporting may have differed depending on the methods used or the publication journal.

The majority of research focused on generic bushmeat hunting (70%), where *bushmeat* is defined as “any non‐domesticated terrestrial mammal, bird, reptile or amphibian harvested for food” (Nasi et al. 2008). The remaining studies focused on targeted hunting of specific species or groups, including ungulates (8%), carnivores (5%), primates (5%), small mammals (5%), birds (4%), and reptiles (2%).

Ninety‐five percent of all studies used interview or questionnaire methodologies to collect data. The remaining 5% of studies used other social science methods. Forty‐six percent of studies used multiple methods to triangulate findings and increase data reliability, including self‐reporting (e.g., hunting or meal diaries [25%], focus group discussions [11%], and observation or hunter follows [18%]).

### Journals and Ethical Human Research Practice

Of the 57 journals reviewed, 27 (47% of all journals) had an ethics policy for research that involved animal subjects, while 21 journals (37%) had an ethics policy for research involving human subjects. Overall, 20 journals (35%) provided policies on both. Broadly, these policies outlined 4 criteria research had to satisfy to be eligible for publication (Table 1). Nineteen journals (33%) required information on these criteria to be included within manuscripts. One journal stated human research ethics should be considered, but did not explicitly state whether authors should document them, while another required authors to assent abidance to an ethical code of conduct upon submission of a manuscript. This code contained detailed stipulations regarding informed consent, the welfare of animal subjects, recognition of local collaborators as coauthors, and the dissemination of research, but no reporting requirements were outlined.

**Table 1:** Ethical reporting requirements of conservation journals (*n* = 57) when research involves human participants

	Number of journals	Percentage of all journals
Requirement documented in author instructions		
include an ethics statement in the manuscript	19	33
follow an ethical code of conduct	16	28
research should be approved by an Institutional Review Board (IRB)	13	23
seek informed consent from participants	12	21
all 4 of above required	6	11
Required information in ethics statement		
include name or name and reference number of IRB	12	21
identify whether informed consent was sought	10	19
identify whether code of conduct was followed	8	14
all 3 of above required	4	7
Location of ethics statement in manuscript		
methods	5	9
separate ethics section	5	9
before the references	2	4
unspecified	7	12

Frequently, we found the policies were inappropriate or used unsuitable terminology. In 5 instances, human participants were referred to as *patients* and ethical requirements were framed in the context of clinical trials, human experimentation, or medical research, rather than conservation, ecological, or zoological research. In addition, 76% of journals that referenced a code of conduct recommended the Helsinki Declaration, a code of practice originally developed for experimental medical research by the World Medical Association in 1964 (Carlson et al. 2004), suggesting guidelines were generic to the publishing house, rather than specifically developed by the journal.

### Upholding of Stated Ethical Principles

The 21 journals that required authors to document ethical safeguards accounted for 122 (66% of all) articles within the review. Yet, only 71 (58%) of these articles documented ethical safeguards, and frequently articles failed to meet journals’ requirements (Table 2). Ten journals stated that research should meet outlined ethical requirements or editors reserved the right not to publish. However, of the 73 articles published in these journals, 45% included no information on research ethics. We found no significant relationship between whether a journal required ethics and the inclusion of ethics within manuscripts (χ^2^ = 1.473, df = 1, *p* = 0.225). The reporting of research ethics did not differ among ecology, zoology, conservation, and interdisciplinary journals, although articles published in journals with a higher impact factor were 0.78 times more likely to require reporting of safeguards (Supporting Information).

**Table 2:** Articles that collected information from human participants about hunting that met journals ethical reporting requirements.*

Ethical reporting requirement	Number of articles published with requirement (% of total)	Number of articles that met requirements (% of total)
Consider ethical safeguards during research	122 (66)	71 (58)
Identify whether research was approved by institutional review board (IRB)	69 (37)	12 (17)
Identify IRB that approved research	69 (37)	6 (9)
Identify whether code of conduct was followed	35 (19)	2 (6)
Identify whether informed consent was sought	31 (16)	17 (46)

^*^Total number of journals examined: 57, total number of articles examined: 185.

### Change over Time

**Figure 2:**
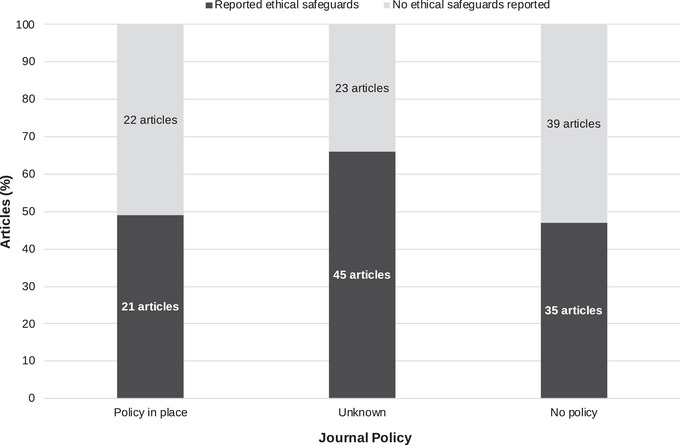
Percentage of peer‐reviewed articles whose authors collected information from local people about hunting activity, categorized by whether the journal had a human research ethics policy in place at the time of publication and whether the article documented ethical safeguards.

**Figure 3:**
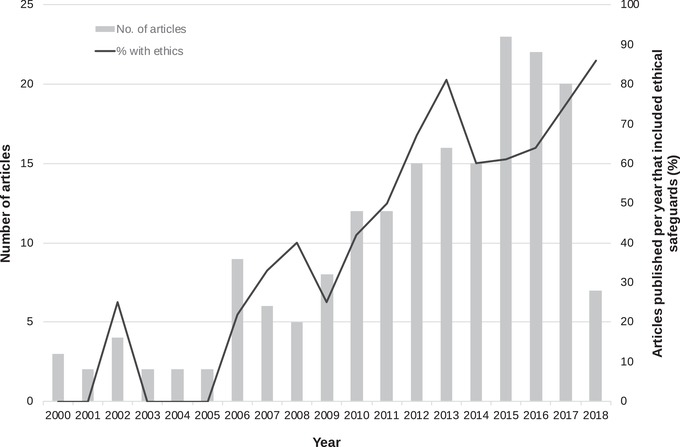
Percentage of articles published from 2000 to 2018 in which authors collected information from local people about hunting and reported the ethical provisions implemented to protect participants.

One factor contributing to this mismatch may be that articles were published before journals introduced their ethics policies. We contacted the editors in chief of the 19 journals in which 2 or more articles were published (*n* = 146, 79% of articles) to ask when policies were introduced. Of the 19 editors contacted, 10 completed the questionnaire (52%) and one declined. On average, editors estimated 36% (minimum 0%, maximum 90%, median 30%) of their editorial boards contained reviewers with social science expertise, and editors believed their journals did either okay (50%) or well (50%) at ensuring only ethically appropriate research was published. Three editors provided information about when ethics guidelines were introduced, and one editor informed us the journal previously had no ethics policy with regard to research that involves people, but they had developed one since receiving our email. For the 43 articles published in years which we know journals had policies in place, nearly 50% failed to include any information on ethical safeguards (Fig. 2). However, we detected an upward trend since 2000 in the proportion of studies published each year that included ethical safeguards (Fig. 3).

### Number of Articles Reporting Ethical Safeguards

Overall, 101 articles (55% of all articles reviewed) included at least one of the 4 ethical safeguards (Table 3). Typically, information on ethics was located in the methods (88% of articles that included ethics), acknowledgements (5%), or both (7%). Of these, only 35 articles (19% of all articles) identified that research had been reviewed and approved by an IRB, based in an academic institution (81% of articles with IRB), government department (18%), or nongovernmental organization (11%). No ethical safeguards were mentioned in 45% of articles. Because we did not contact authors directly, we were unable to discern whether ethics were considered and implemented during research but not reported in articles (reporting bias) or whether they were omitted from the research process altogether (poor research practice).

**Table 3:** Types of ethical safeguards documented in hunting articles (*n*=185)

Instituitional Review Board approval	Informed consent	Anonymity or confidentiality	Code of conduct followed	No. of articles (%)
✓	✓	✓	✓	1 (<1)
✓	✓	‐	✓	1 (<1)
	✓	✓	✓	3 (2)
‐	✓	‐	✓	3 (2)
✓	‐	‐	‐	8 (4)
‐	‐	✓	‐	12 (6)
✓	✓	‐	‐	12 (6)
✓	✓	✓	‐	13 (7)
‐	✓	✓	‐	22 (12)
‐	✓	‐	‐	26 (14)
**35 (19)**	**81 (44)**	**51 (28)**	**8 (4)**	**Totals** *
No ethical considerations documented				84 (45)

^*^55% of studies reviewed documented at least one ethical safeguard, ( ) = %

### Types of Ethical Safeguards Documented by Authors

Eighty‐one (44%) of all articles reported seeking participant consent (Table 3); of articles with consent described Twenty‐six articles reported securing consent verbally (32% of articles that documented consent), 3 articles (4%) secured written consent, and 3 articles (4%) identified that written consent would be inappropriate due to high illiteracy levels. Consent was typically sought from individual participants, but occasionally consent was obtained at the community level (e.g., from the village chief). However, whether subsequent consent was also sought from participating individuals was not always clear.

When discussing consent, authors rarely used the same terminology. In 5 articles, consent was described as “free, prior and informed” (Table 4). In 16 articles (20% of those that reported seeking participant consent), authors stated respondents consented to participate voluntarily (free consent) and used phrases such as “interviews only proceeded with respondents’ permission,” or “respondents were told they could skip any uncomfortable questions or could abort interviews at any time.” In 19 articles (24%), authors explained the research purpose to participants before seeking consent (informed consent). Few articles described explaining potential risks of participation. Consent was described as both free and informed in a further 31 articles (39%). In 10 articles (12%), consent was sought but no indication of whether consent was free or informed was provided.

**Table 4:** Ways in which the consent process was described in hunting studies (*n* = 81)

Description	Number of articles (%)
Free, prior, and informed consent	5 (6)
Information given to participant before consent sought (informed consent)	19 (24)
Respondents participated voluntarily (free consent)	16 (20)
Consent was both free and informed	31 (39)
No information on how consent was sought was provided	10 (12)

### Anonymity, Confidentiality, and Research Sensitivity

Anonymity was reported as assured in 44 articles (24%), 11 articles (6% of all articles) documented offering respondents’ confidentiality, and 4 articles (2%) reported both. Anonymity was typically afforded at the individual or household level, either by not recording names or by anonymizing data after collection. Six articles (3%) suggested respondents would be more inclined to reveal the truth about sensitive or illegal hunting behavior if responses were anonymous or confidential. In 5 articles (3%), authors also anonymized village locations to afford communities greater protection from potential reprisals. However, in 86 articles (46%), the specific village or location where research was conducted was either named, co‐ordinate referenced, or identified on a map. In several instances, authors provided individual estimates of each community's hunting activity. In one article, the author assured village‐level anonymity, yet they had previously published research from the same site in which villages were named.

In 129 articles (70% of all articles), hunting wildlife was an illegal or restricted activity, in a further 32 articles (17%), the status of hunting was undescribed (Table 5). Nearly all these articles required participants to self‐report their hunting activity (*n* = 151, 94%), yet ethical safeguards were only documented in 58% of these articles (n=94) (Table 5).

**Table 5:** Number of articles in which authors reported different ethical safeguards implemented to protect human participants when researching different types of hunting legality

	Hunting illegal or conditional (%)	Hunting legal (%)	Legality of hunting unknown (%)	Total number of studies (%)
Total number of articles	129 (70)	24 (13)	32 (17)	185 (100)
Articles reporting ethics	79 (61)	7 (29)	15 (47)	101 (55)
Ethical consideration reported:				
consent	62 (48)	6 (25)	13 (41)	81 (44)
confidentiality and/or anonymity,	48 (37)	1 (4)	2 (6)	51 (28)
institutional review board approval	30 (23)	1 (4)	4 (13)	35 (19)
code of conduct followed	6 (3)	0 (0)	2 (6)	8 (4)
all 4 considerations	1 (<1)	0	0	1 (<1)

### Adoption of Specialized Research Methods

During the review, we identified 18 articles (10% of all articles) in which authors reported using specific methods to afford respondents greater anonymity and to minimize biases (e.g., underreporting due to social desirability bias) associated with asking questions about sensitive behaviors. Seven articles reported asking respondents about hunting indirectly, either by asking questions about community level (rather than individual) hunting or by encouraging respondents to reply in the third person to prevent implication in illicit activity. In one article, choice experiments were used to assess respondent's willingness to hunt under different livelihood and law enforcement scenarios. In another article, questions were asked in a group setting. In a third article, fuzzy‐logic cognitive mapping was used in a group setting to reduce the reliance on individual reporting. In 8 articles (4%), authors documented using the randomized response technique (RRT) (4 articles) or the unmatched count technique (UCT) (4 articles), both of which are methods designed to assure respondents’ greater anonymity. However, to triangulate results and test method reliability, respondents were also asked directly about hunting activity. Several authors reviewed the merits of UCT and RRT, but deemed them inappropriate or inapplicable methods for their research context.

## Discussion

Social science methods are increasingly applied in conservation research (Bennett et al. 2016; Young et al. 2018). Within our sample, we documented a rise since 2010 in the number of studies using interviews to research hunting, and an increase in the proportion of articles published per year that featured ethical safeguards, suggesting reporting of ethics is becoming more common. However, we often found too few safeguards were documented per article. Just one study met all 4 of our ethical criteria, and only 9% of studies listed at least 3 safeguards.

Frequently, safeguards were poorly described; thus, we lacked sufficient detail to determine the quality of the safeguards implemented. Of the studies that listed consent, many failed to identify whether consent was informed. We noted inconsistency in how consent was sought and from whom. Best‐practice guidelines such as those of the International Society of Ethnobiology Code of Ethics recommend seeking consent at both individual and population levels. Yet often consent was only identified as sought at the individual or community level. Although mechanisms such as communal consent may be most suitable in strongly hierarchical societies, used in isolation, they can undermine individuals’ freedom to opt in or out of research. Consequent participation may reflect social obligation, rather than genuine desire to be included. The absence of a shared vocabulary, alongside poor acknowledgment and discussion of these types of ethical issues within articles, may reflect insufficient ethics training and highlights a pressing need for capacity building and the mainstreaming of research ethics practice throughout the conservation sector.

### Adequately Assuring Anonymity

Asking local people, often in contexts where power imbalances prevail, to report sensitive behaviors without asking if they desire anonymity is ethically questionable. Anonymity can help build participant trust, enhance receptivity to questioning, and increase honest reporting (Ong & Weiss 2000). Yet, anonymity was rarely referenced in articles. Some articles documented using indirect questioning techniques such as RRT and UCT to assure greater anonymity (Nuno & St. John 2015); however, these methods were always employed alongside direct questioning. Although this is necessary to verify the robustness of estimates, it undermines any additional protection as participants are still required to directly identify their behavior. Although we welcome the addition of these techniques to conservation's methodological toolbox, they do not present a panacea for overcoming ethically challenging aspects of research. Additional research is required to ascertain how these methods are best employed.

Research locations were identified either by name or coordinates in nearly half of all hunting studies. Although identifying study sites appears to be a common practice in conservation, we, along with others, urge greater caution when doing so (Solomon et al. 2016; St. John et al. 2016). If results for sensitive activities are publicly reported at the community level (e.g., by village name), communities may be subject to direct reprisals. It can also undermine any assurances of anonymity, especially if research is conducted in communities where households are few or participants are easily identifiable. Adopting community pseudonyms in publications and not identifying research locations on maps are precautionary measures authors can implement in publications to better safeguard participants from reprisals (Solomon et al. 2007, 2016; St. John et al. 2016). However, achieving this may pose a challenge because identifying locations is often necessary to enable meaningful interpretation of results (St. John et al. 2016). If anonymity is not possible to assure, we encourage researchers to report why this is so, alongside alternate measures implemented to protect research participants.

Within our sample, we encountered authors who presumably published multiple articles from one research project. In earlier articles communities were named, but in more recent publications communities were anonymized. This change in approach may result from different journal requirements or perhaps signify a shift toward greater ethical awareness. Regardless, this highlights that researchers should be aware that once published research cannot be redacted; thus, thorough consideration of ethical implications must occur before publication.

### Ethical Exclusion

Nearly half of the articles (45%) reviewed failed to report human research ethics. Although this does not suggest these studies failed to employ ethical safeguards, it raises questions about the ethical integrity of research. As an inherent aspect of social research design, we argue ethical safeguards should always be reported in peer‐reviewed articles. A principal purpose of published literature is to inform future research design. Yet, if publications fail to reference research ethics, how can researchers learn to strengthen future research ethics and practice? Although identifying the cause of this exclusion was beyond the scope of our review, word limits and author or reviewer oversight are 2 plausible causes. Some of these challenges can easily be overcome by, for example, placing ethical‐approval references in acknowledgments, which may be excluded from word counts, and by providing greater guidance to authors and peer reviewers on ethics reporting. Requiring the routine submission of research protocols, either as appendices or in open‐access repositories, alongside manuscripts would enable readers to learn from previous researchers, ensure appropriate ethical procedures were met, and promote greater transparency in the research process.

Given the medical origins of ethical review (Carlson et al. 2004; Wiles 2013), we acknowledge that procedural measures outlined by IRBs are not always appropriate or attainable in conservation contexts. Generally, however, there is agreement that ethical review protects participants and promotes research transparency (Vanclay et al. 2013), and formal ethical review is increasingly becoming a mandatory and legal prerequisite of governments, funders, and institutions (Dyer & Demeritt 2009; Speiglman & Spear 2013). Although there is a tendency for ethical review processes to be viewed as a bureaucratic box ticking exercise (Dyer & Demeritt 2009; Lunn 2014), procedural ethics offer researchers a vital checklist of ethical factors to consider (Guillemin & Gillam 2004). As researchers are increasingly required to secure IRB approval, we urge researchers to document this, along with the specific safeguards implemented in manuscripts. Not only does this strengthen the quality of research, but it also removes the ability of readers to doubt the ethical integrity of research and, thus, conservation as a sector.

### Responsibility for Reporting

If researchers are responsible for conducting ethical research, then responsibility for publishing research of ethical rigor lies along all stages of the publication chain (St. John et al. 2016; Teel et al. 2018). As gatekeepers to publication, journals have power to ensure research is ethically conducted, and that only ethical research is published. However, to do so requires transparent and explicit ethics policies. Yet within our sample, only 37% of journals outline the ethical measures they expect authors to abide by or include in manuscripts if research involves people, and where journals provided information on research ethics, it was sometimes inappropriate. Although our results suggest journals are performing better than 20 years ago when a similar study found only 3% of conservation or ecology, wildlife, and zoology journals issued instructions to authors regarding human research ethics (Marsh & Eros 1999), there is still significant scope for improvement.

One immediate way to rectify this is for journals to review and revise their ethics policies and the instructions issued to authors. Journals must provide researchers with fair, equitable, and explicit best practice guidelines. These must be relevant to conservation contexts and should clearly outline the basis for rejection. Some examples exist (Oryx 2001; Wilmé et al. 2016); but these could be developed further by explicitly identifying reporting standards in a way similar to Freckleton (2018) for the publication of code. The inclusion of ethics statements in manuscripts should be mandatory. Statements should identify whether research was approved by an ethical review board (IRB) or if a code of conduct was followed; the process of informed consent; whether safeguards, such as anonymity or confidentiality, were in place; alongside discussions of any ethical challenges encountered during research. Authors should be supported to achieve this through the provision of sufficient word space. In addition, to promote transparency and accountability, journals should encourage the publication of research protocols as appendices. However, in recognition of the fact that much conservation research is conducted by authors from institutions governed by different rules, research standards, and ethical expertise (St. John et al. 2016), we warn against the adoption of blanket policies that only enable authors with IRB clearance to publish, as this may exclude researchers based in small underresourced organizations (St. John et al. 2016).

We found journal stipulations did not guarantee compliance; ethics were omitted in over 40% of articles where ethics were supposedly required. One reason may be that articles were published after guidelines were introduced, however, of the journals that could be assessed we found 50% of articles published after guidelines were introduced still failed to include ethical safeguards. Our results highlight a significant mismatch between journals’ stipulations and their publication practice suggesting, at least within our sample, that journals are failing to comply with their own standards with regard to research that involves human subjects. If conservation journals intend to increase publication of high‐quality social science (Teel et al. 2018), they must ensure all manuscripts are scrutinized with appropriate levels of ethical rigor, and in accordance with published journal policy. To achieve this, journal editorial boards must be composed of those with adequate expertise to properly review social science research (Campbell 2005) and those reviewing research must adhere to the guidelines of the journal and ensure manuscripts adequately document ethical considerations. Only research that meets the high‐quality standards outlined by the journal should be published (St. John et al. 2016).

### Review Limitations

We reiterate that our review by no means represents a comprehensive assessment of the ethical provisions implemented to protect participants by conservationists during research and that our findings do not suggest conservation researchers are failing to act ethically. However, they do demonstrate that reporting of ethical safeguards during hunting research has been poor. We acknowledge that we did not discuss many important ethical issues such as data security and storage, the role of power dynamics in research, or how ethical safeguards should be extended to others involved in research, such as research assistants. However, rarely did we encounter discussions of these issues in manuscripts. Given the poor coverage of even the most basic ethical safeguards in articles, we believe highlighting the absence of these fundamentals was a critical first step.

In addition, our review focused only on academic literature. Yet unpublished, practitioner‐generated research represents a considerable portion of hunting literature. Although human research ethics are increasingly recognized by conservation organizations (e.g., IIED 2018), many of them do not have institutional structures in place to review research that involves people. Considering the deficit of ethical reporting we found in articles from authors based in academic institutions where IRB is the norm, we find this concerning.

Recent research suggests that early‐career conservation scientists receive little, if any, mandatory training in the philosophy or ethics of conservation and research (Saltz et al. 2018). Unlike research that involves animals (Costello et al. 2016), few guidelines exist specifically addressing the unique ethical challenges of conducting research with human subjects in conservation. As interdisciplinarity grows and social science becomes further ingrained in the fabric of conservation research, conservationists must be provided with adequate ethical training to ensure they recognize ethical issues and are properly equipped to negotiate them. Furthermore, as a discipline, conservation science should strive to develop a code of ethics for human research, this should seek to promote good ethical practice and provide guidance to researchers on how to navigate the complex contexts in which conservation research is conducted. Adhering to rigorous ethical standards should be viewed as an investment that not only strengthens research practice and integrity, but also secures greater engagement and buy‐in of participants.

## Supporting information

The full review protocol (Appendix S1), the questionnaire sent to editor in chiefs (Appendix S2), a list of all journals featured in the review (Appendix S3), a list of all articles reviewed (Appendix S4), and additional analyses (Appendix S5) are available as part of the on‐line article. The authors are solely responsible for the content and functionality of these materials. Queries (other than absence of the material) should be directed to the corresponding author.
