# Supplementary material for: Conservation publications and their provisions to protect research participants
Source: Conserv Biol. 2019 Jun 14;34(1):80–92. doi: 10.1111/cobi.13337 (PMC7028057; doi:10.1111/cobi.13337)
Supplement: Supplementary file 1 — The full review protocol (Appendix S1), the questionnaire sent to editor in chiefs (Appendix S2), a list of all journals featured in the review (Appendix S3), a list of all articles reviewed (Appendix S4), and additional analyses (Appendix S5) are available as part of the on‐line article. The authors are solely responsible for the content and functionality of these materials. Queries (other than absence of the material) should be directed to the corresponding author. [file COBI-34-80-s001.docx]

# Conservation publications and their provisions to protect research participants

# Electronic Supplementary Materials

**Contents**

Appendix S1 – Review Protocol……………………………………………………. 1

Appendix S2 – Email questionnaire sent to Editors of journals…………………. 6

Appendix S3 – List of journals featured in review………………………………… 9

Appendix S4 – List of articles featured in review…………………………………. 12

Appendix S5 – Additional Analyses………………………………………………… 28

# Appendix S1 - Review Protocol

## Step One – Conducting systematic searches & compiling results

Between 15^th^ August 2017 and 15^th^ May 2018, we conducted searches in three databases:

- Google Scholar
- Scopus
- Web of Science™

In Scopus and Web of Science, we searched the term:

*“hunting OR wildlife OR hunter OR bushmeat OR wild meat OR poaching OR poacher AND interview”*

Using the following criteria:

- Year: 2000 – 2018
- Category:  Ecology or Zoology or Biodiversity Conservation
- Document type: Article
- Language: English

Due to the inability to directly export search results from Google Scholar, we used Harzing’s Publish or Perish software to search for and extract the first 1000 article titles (Harzing 2007). We conducted searches on a year by year basis using all of the words: ‘interview, hunting, wildlife’, and any of the words ‘hunter, bushmeat, wild meat, poaching, poacher’ on a year by year basis. All search results were downloaded as .csv files or .text files, and imported into Microsoft Excel. Here, results were consolidated into one spreadsheet.

Total records found: 4456

## Step Two – Title Scan

Using the deduplicate function in Excel, all articles identified more than once were removed. All titles were then scanned by both HI and SB, using the following criteria:

Does the title suggest the study is about hunting?

- If yes 🡪 accept
- If no 🡪 reject

Both authors scanned all titles and Cohen’s Kappa statistic was used to assess for inter-agreement, with a result of 0.67 suggesting substantive agreement (Watson & Petrie 2010). We adopted a precautionary approach, whereby if one author but not the other accepted a title we pushed the article through to the next stage. Results:

Accepted: 626

Rejected: 3830

Duplicates: 231

## Step Thee – Abstract Scan

After the title scan, we read the abstracts of 626 articles in full. Articles were randomly assigned between HI and SB, and reviewed against the following criteria:

- Does the study research wildlife hunting by local communities?
- Does the study use interviews, questionnaires, focus groups, hunter diaries or hunter follows to collect data?
- Was the research conducted in any country located in Africa, Central America, South America, South East or Southern Asia, or the Pacific islands?
- Does the article assess hunting of terrestrial species only?

If studies met all four criteria they were accepted. A precautionary approach was employed, with authors peer-reviewing each others work after 20 and 100 articles to ensure agreement. Results:

Accepted: 227

Rejected: 371

Duplicates: 28

## Step Four – Data extraction

Each study was randomly allocated to HI or SB for full review. Data was extracted on:

- The journal the article was published in
- The year the article was published
- The year/s the research was conducted
- The country the research was conducted in
- The location and type of institution of the lead author
- The species of research interest
- The legal status of hunting in the research area (illegal, legal, conditional, unknown)
- The formal designation of the study site (Protected Area, Unprotected Area, Unknown)
- The main habitat type (forest, savannah, wetland etc)
- The research methods used
- Whether these methods asked respondents directly or indirectly about hunting activity
- Whether the author included any ethical considerations (Yes / No), determined when one or more of the following were included:
  1. The research was formally approved by an Institutional Review Board (Yes / No)
     1. If yes, the type of review board (government, institution, NGO, unknown) and whether the IRB reference was included in the manuscript (Yes / No)
  2. Whether the research mentioned consent, determined when one or more of the following were mentioned:
     1. Free, Prior, Informed Consent (FPIC)
     2. That respondent’s participation in the study was voluntary, or that the respondent voluntarily has permission to conduct research and record responses (Free consent)
     3. That information about the research was provided to respondents before research took place (Informed consent)
  3. That the identity of research participants, or communities would remain anonymous (anonymity), and /or that any information provided would remain private (confidential)
  4. Whether the article mentioned whether research followed a specific ethical Code of Conduct (Yes / No)
- Where ethical considerations were listed in the manuscript (Methods, Acknowledgments, Ethics Section)
- Whether the research acknowledged the use of Research Assistants during data collection (Yes, No, unknown)
- We also made specific notes or comments about other considerations or inconsideration we came across in each manuscript. This included whether the authors named or identified study locations in the article (Yes / No).

Five further duplicates were identified and removed, 34 studies were identified as irrelevant (for example, they modelled offtake), 2 articles were non-English and 1 article was inaccessible. In total 185 articles were reviewed in full.

Alongside the data extraction process, each article was imported into NVivo. Each article was then reviewed, with text coded according to specific themes developed apriori by both authors. The purpose of undertaking this qualitative aspect of the review was to gain a more robust understanding of the different issues discussed by authors and the measures implemented by authors. Apriori codes were:

- Anonymity: any text highlighting or refering to anonymity
- Confidentiality: any text referring to confidentiality
- Consent: text which discussed the methods used to obtain consent
- Field assistants: any information about who collected the data, how they were selected/recruited and their qualifications and/or training
- Official approval: any official approval or permits sought to conduct data, includes IRB review, country research permits
- Payment: whether any payment or compensation was offered to respondents
- Research methods: the types of methods used, the reason for their selection, and their utilty
- Sensitivity: whether research was sensitive in any way, whether any measures were put in place to account for thse

Table S1a. Article selection process. Papers were discarded when duplicates, irrelevant, unable to access, or in a foreign language.

| **Stage** | | **n** |
| --- | --- | --- |
| 1. Identification | GScholar, Scopus & WoS searches | 4456 |
| 1. Eligibility | Title screening | 626 |
|  | Title & Abstract reviewed | 227 |
| 1. Extraction | Full article review & data extraction | 185 |
| 1. Analysis |  | 185 |

Figure S1a. Flow diagram of the review process from data compiling to data extraction.

# Appendix S2 – Email questionnaire sent to Editor-in-Chiefs of featured journals

**Introduction**

We are currently conducting a literature review of the ethical considerations towards human research participants listed by authors when publishing articles. Specifically, our review focuses on studies that use social science methods such as interviews to investigate hunting of wildlife by local communities.

The three aims of our review are to:

(1) Review the guidelines journals offer to authors, and the ethical standards required by journals to be met prior to publication.

(2) Describe the types of safeguards and ethical considerations documented by authors to protect human research participants.

(3) Infer, based on the information about ethical procedures included in articles, the extent to which conservation studies adhere to good ethical practice.

To achieve the first aim we have reviewed the Guidelines to Authors that journals provide regarding human research ethics. We are contacting journal Editors-in-Chief directly, to ask for their perspectives and to request specific information about human research ethics and how journals ensure compliance when reviewing and publishing conservation articles.

As the Editor-in-Chief of a journal featured within our review sample, we invite you to complete our short questionnaire.

**Informed Consent**

Before we start, we must obtain your consent. Please read through the terms below before agreeing to participate by ticking the ‘yes’ box below. Please ask any questions before taking part by contacting the researchers directly.

You will be asked to answer a few short questions on the policy of the journal regarding human research ethics. The questionnaire should take no more than 15 minutes.

Your participation is voluntary. All questions are optional. Where appropriate we have included a ‘Prefer not to say’ option. You may withdraw at any point during the questionnaire for any reason, before submitting your answers, by closing the browser.

As part of the survey we require your name and the name of the journal you edit. We will use all reasonable endeavours to keep your answers confidential. Any personal information that could identify you will be removed or changed before files are shared with other researchers or results are made public. At no point will you as individual, or the journal for which you edit, be named in association with the responses you provide.

The information you provide will be stored in a password-protected file and may be used in academic publications. Your IP address will not stored. Research data will be stored for a minimum of three years after publication or public release.

Before preparing this questionnaire, the University of Oxford Central University Research Ethics Committee was consulted regarding the need to secure formal ethical approval. This project was approved for continuation without formal ethical clearance.

If you have any concerns, please speak to the researchers [NAMES & CONTACTS PROVIDED], who will do their best to answer your query. The researcher should acknowledge your concern within 10 working days and give you an indication of how they intend to deal with it.

If you have read the information above and agree to participate with the understanding that the data you submit will be processed accordingly, please check the relevant box below to get started.

Yes, I agree to take part

No, I do not wish to participate

**About you**

We require this information so we can match the responses with the specific journal. Your name and email will not be shared.

| Your full name |
| --- |
| Your email |
| The name of the journal which you edit |
| Your Editorial position |
| For how many years have you been editing this journal? |
| Do you have any training (formal or other) in the social sciences?  Yes / No |
| If yes, please describe your training |

**About the journal**

| Please describe (as briefly as possible) the journal's peer-review process |
| --- |
| In your opinion, what proportion of the journal's editorial board are trained social scientists?  0% / 10% / 20% / 30% / 40% / 50% / 60% / 70% / 80% / 90% / 100% |

**Journal policy on human research ethics**

| Does your journal have a formal policy, or set of guidelines/standards with regards to human research ethics?  Yes / No / Not Sure |
| --- |
| In what year were these introduced? |
| Please could you describe the processes used to ensure authors submitting manuscripts comply with the journal's guidelines/policies on human research ethics? |
| When publishing papers, please could you describe the processes used to ensure peer-reviewers & editors comply with the journal's guidelines/policies on human research ethics? |
| Overall, how well do you think your journal does at ensuring only ethical research is published?  Very badly / badly / OK / well / Very well |
| What do you perceive as the biggest challenge when ensuring only ethically robust social research is published? |

**Follow up Questions**

| If we require further clarification of your response, please may we get in touch further?  Yes / No |
| --- |
| Once published, would you like to receive a copy of the article?  Yes / No |
| If you have any specific comments, feedback or questions, please feel free to use the space below. |

# Appendix S3 - Journals

**Table S3a.** Journals in which reviewed articles were most frequently published (n=185)

| Journal | No. of studies | % of sample |
| --- | --- | --- |
| Oryx | 33 | 18 |
| Biological Conservation | 23 | 12 |
| Conservation Biology | 18 | 10 |
| Ecology & Society | 12 | 6 |
| Tropical Conservation Science | 11 | 6 |
| Other | 88 | 48 |

**Table S3b.** List of journals that published articles featured in the review (n=57)

| Journal | Publisher | n |
| --- | --- | --- |
| African Journal of Ecology | Wiley | 3 |
| African Journal of Wildlife Research | BioOne | 3 |
| African Study Monographs | Kyoto University | 2 |
| African Zoology | Taylor & Francis | 1 |
| American Journal of Primatology | Wiley | 2 |
| Animal Biodiversity & Conservation | Museu de Ciències Naturals de Barcelona | 1 |
| Animal Conservation | Wiley | 5 |
| Athens Journal of Tourism* | Athens Institute for Education & Research | 1 |
| Biodiversity & Conservation | Springer | 5 |
| Biological Conservation | Elsevier | 23 |
| Chelonian Conservation & Biology | Chelonian Society | 1 |
| Conservation and Society | Medknow Publications | 6 |
| Conservation Biology | Wiley | 18 |
| Deviant Behavior* | Taylor & Francis | 1 |
| Diversity and Distributions | Wiley | 1 |
| EcoHealth | Springer | 1 |
| Ecological Economics | Elsevier | 2 |
| Ecology and Society | Open Access Publishing | 12 |
| Environment, Development & Sustainability | Springer | 1 |
| Environmental Conservation | Cambridge University Press | 7 |
| Environmental Management | Springer | 2 |
| Ethiopian Journal of Environmental Studies and Management | Bahir Dar University, Ethiopia | 1 |
| European Journal of Wildlife Research | Springer | 1 |
| Folia Primatologica | Karger | 1 |
| Herpetological Conservation and Biology | Independent | 1 |
| Human Dimensions of Wildlife | Taylor & Francis | 3 |
| Human Ecology | Springer | 3 |
| HYATI Journal of Biosciences | Bogor Agricultural University (IPB) | 1 |
| Integrative Zoology | Wiley | 1 |
| International Journal of Business and Social Science* | Centre for Promoting Ideas, USA | 1 |
| International Journal of Comparative and Applied Criminal Justice* | Taylor & Francis | 1 |
| International Journal of Conservation Science | Alexandru Ioan Cuza University Publishing House | 1 |
| International Journal of Mol. Ecol. and Conserv | BioPublisher | 1 |
| International Journal of Primatology | Springer | 1 |
| Revista de Biología Tropical | Universidad de Costa Rica | 1 |
| Journal for Nature Conservation | Elsevier | 4 |
| Journal of Applied Ecology | British Ecological Society | 1 |
| Journal of Biodiversity & Endangered Species | OMICS International | 1 |
| Journal of Ethnobiology and Ethnomedicine* | BioMedCentral | 1 |
| Journal of Ethnobiology* | Society of Ethnobiology | 1 |
| Journal of Horticulture and Forestry | Academic Journals | 1 |
| Journal of Natural Sciences Research | American Research Institute for Policy Development | 1 |
| Journal of Tropical Biology and Conservation | Universiti Malaysia, Sabah | 1 |
| Journal of Tropical Ecology | Cambridge University Press | 1 |
| Malaysian Applied Biology | Universiti Kebangsaan Malaysia | 1 |
| Natural Resources | Scientific Research Publishing | 2 |
| Obeche Journal | NA | 1 |
| Oryx | Cambridge University Press | 33 |
| PARKS | IUCN World Commission on Protected Areas | 1 |
| PLoS One* | PLoS One | 1 |
| Regional Environmental Change | Springer | 2 |
| Revue d’Ecologie (Terre et Vie) | Société Nationale de Protection de la Nature et d’Acclimatation de France | 1 |
| The Asian International Journal of Life Sciences | Rushing Water Publishers Ltd | 1 |
| Tropical Conservation Science | Sage | 11 |
| Vie et Milieu | Observatoire Océanologique - Laboratoire Arago - Université Pierre et Marie Curie | 1 |
| Wildlife Research | CSIRO Publishing | 1 |
| Wildlife Society Bulletin | Wiley | 1 |

*Titles marked with an * represent journals for which zoology, ecology or conservation is not the primary focus*

# Appendix S4 - Articles (n=185)

**Table S4a.** Countries where studies were conducted. 1 study was conducted in two countries (Kenya and Tanzania), and one study was conducted in multiple countries within the global south, and thus was excluded from this table. Rows highlighted in grey represent countries purposefully excluded from the review.

| **Country** | **No. of studies** | **No. of studies where author based at an in-country institution** | **No. of studies conducted in -country & by author based at an in-country institution** | |
| --- | --- | --- | --- | --- |
|  |  |  | **n** | **%** |
| Argentina | 1 | 0 | 0 | 0% |
| Bangladesh | 2 | 1 | 1 | 50% |
| Belize | 2 | 0 | 0 | 0% |
| Bolivia | 3 | 1 | 1 | 33% |
| Botswana | 1 | 0 | 0 | 0% |
| Brazil | 19 | 14 | 13 | 68% |
| Cambodia | 2 | 0 | 0 | 0% |
| Cameroon | 10 | 3 | 2 | 20% |
| Central African Republic | 2 | 0 | 0 | 0% |
| China | 5 | 3 | 3 | 60% |
| Columbia | 1 | 0 | 0 | 0% |
| Costa Rica | 2 | 2 | 2 | 100% |
| Cote D'Ivoire | 0 | 1 | 0 | NA |
| Demo. Rep. of Congo | 2 | 0 | 0 | 0% |
| Ecuador | 6 | 3 | 1 | 17% |
| Equatorial Guinea | 9 | 0 | 0 | 0% |
| Gabon | 4 | 0 | 0 | 0% |
| Ghana | 4 | 1 | 1 | 25% |
| Global South | 1 | 0 | 0 | 0% |
| Guinea-Bissau | 1 | 1 | 1 | 100% |
| Guyana | 2 | 0 | 0 | 0% |
| Honduras | 2 | 0 | 0 | 0% |
| India | 5 | 2 | 2 | 40% |
| Indonesia | 7 | 2 | 0 | 0% |
| Iran | 3 | 1 | 1 | 33% |
| Ivory Coast | 1 | 0 | 1 | 100% |
| Kenya | 3 | 2 | 2 | 67% |
| Liberia | 1 | 1 | 1 | 100% |
| Madagascar | 9 | 1 | 1 | 11% |
| Malaysia | 3 | 2 | 1 | 33% |
| Mexico | 5 | 5 | 5 | 100% |
| Mozambique | 1 | 0 | 0 | 0% |
| Myanmar | 4 | 0 | 0 | 0% |
| Namibia | 3 | 0 | 0 | 0% |
| Nepal | 1 | 1 | 1 | 100% |
| Nigeria | 8 | 8 | 8 | 100% |
| Niue | 1 | 0 | 0 | 0% |
| Peru | 4 | 0 | 0 | 0% |
| Philippines | 1 | 0 | 0 | 0% |
| Samoa | 1 | 0 | 0 | 0% |
| Sao Tome | 2 | 0 | 0 | 0% |
| Sierra Leone | 3 | 0 | 0 | 0% |
| South Africa | 3 | 6 | 3 | 100% |
| Taiwan | 1 | 0 | 0 | 0% |
| Tanzania | 24 | 4 | 4 | 17% |
| Thailand | 1 | 1 | 0 | 0% |
| Uganda | 3 | 0 | 0 | 0% |
| USA | 0 | 37 | 0 | NA |
| Venezuela | 1 | 1 | 0 | 0% |
| Vietnam | 3 | 1 | 1 | 33% |
| Western Guinea | 1 | 0 | 1 | 100% |
| Zimbabwe | 3 | 0 | 0 | 0% |
| Australia | - | 7 | - | NA |
| Canada | - | 2 | - | NA |
| Denmark | - | 6 | - | NA |
| France | - | 1 | - | NA |
| Germany | - | 2 | - | NA |
| Italy | - | 3 | - | NA |
| Japan | - | 2 | - | NA |
| New Zealand | - | 3 | - | NA |
| Norway | - | 3 | - | NA |
| Portugal | - | 3 | - | NA |
| Singapore | - | 3 | - | NA |
| Spain | - | 4 | - | NA |
| The Netherlands | - | 2 | - | NA |
| UK | - | 38 | - | NA |
| Unknown | - | 1 | - | NA |

**List of articles featured in the review (n=185)**

1. Abere SA, Lateef FL, Lameed GA. 2016. Assessment of Hunters and Other Rate of Illegal Activities in Afi-Mbe-Okwango Division, Cross River State, Nigeria. Natural Resources **07**:287–294. Available from <http://www.scirp.org/journal/doi.aspx?DOI=10.4236/nr.2016.75025>.
2. Abram NK et al. 2015. Mapping perceptions of species’ threats and population trends to inform conservation efforts: the Bornean orangutan case study. Diversity and Distributions **21**:487–499. Available from <http://doi.wiley.com/10.1111/ddi.12286>.
3. Aisher A. 2016. Scarcity, Alterity and Value: Decline of the Pangolin, the World’s Most Trafficked Mammal. Conservation and Society **14**:317–329. Available from <http://www.conservationandsociety.org/text.asp?2016/14/4/317/197610>
4. Aiyadurai A, Singh NJ, Milner-Gulland E. 2010. Wildlife hunting by indigenous tribes: a case study from Arunachal Pradesh, north-east India. Oryx **44**:564–572. Available from <https://doi.org/10.1017/S0030605309990937>
5. Akani GC, Petrozzi F, Ebere N, Dendi N, Phil-eze P, Nioking A, Luiselli L. 2015. Correlates of indigenous hunting techniques with wildlife trade in bushmeat markets of the Niger delta (Nigeria). Life and Enviornment **65**:169–174. Availble from <https://www.researchgate.net/publication/285209754_Correlates_of_indigenous_hunting_techniques_with_wildlife_trade_in_bushmeat_markets_of_the_Niger_delta_Nigeria>
6. Akinyemi AF, Kayode IB. 2010. Impact of Human Activities on the Distribution of Ungulates in Old Oyo National Park, Nigeria. Obeche Journal **28**:106–111. Availble from <https://www.researchgate.net/publication/235788244_Impact_of_Human_Activities_on_the_Distribution_of_Ungulates_in_Old_Oyo_National_Park_Nigeria>
7. Alexander JS, McNamara J, Rowcliffe JM, Oppong J, Milner-Gulland E. 2015. The role of bushmeat in a West African agricultural landscape. Oryx **49**:643–651. Available from <http://www.journals.cambridge.org/abstract_S0030605313001294>.
8. Allebone-Webb S, Kumpel NF, Rist J, Cowlishaw G, Rowcliffe J, Milner-Gulland E. 2011. Use of Market Data to Assess Bushmeat Hunting Sustainability in Equatorial Guinea. Conservation Biology **25**:597–606. Available from <http://doi.wiley.com/10.1111/j.1523-1739.2011.01681.x>.
9. Altrichter M. 2006. Wildlife in the life of local people of the semi-arid Argentine Chaco. Biodiversity and Conservation **15**:2719–2736. Available from <http://link.springer.com/10.1007/s10531-005-0307-5>.
10. Altrichter M, Almeida R. 2002. Exploitation of white-lipped peccaries Tayassu pecari *(Artiodactyla: Tayassuidae)* on the Osa Peninsula, Costa Rica. Oryx **36**:126–132. Available from <https://doi.org/10.1017/S0030605302000194>
11. Apaza L, Wilkie D, Byron E, Huanca T, Leonard W, Pérez E, Reyes-García V, Vadez V, Godoy R. 2002. Meat prices influence the consumption of wildlife by the Tsimane’ Amerindians of Bolivia. Oryx **36**:382–388. Available from <http://www.journals.cambridge.org/abstract_S003060530200073X>.
12. Ariya G. 2015. Wildlife Snaring by the Local Community in Ruma National Park, Kenya: Can Conservation Tourism be an Alternative Livelihood Strategy? International Journal of Business and Social Science **6**:141–149. Available from <http://ijbssnet.com/journals/Vol_6_No_2_February_2015/19.pdf>
13. Ashayeri S, Newing H. 2012. Meat, Markets, Pleasure and Revenge: Multiple Motivations for Hunting in Bamu National Park, Fars Province, Iran. Parks **18**:125. Available from <https://cmsdata.iucn.org/downloads/parks_issue_18_1_low_resolution_file.pdf#page=127>.
14. Azhar B, Lindenmayer D, Wood J, Fischer J, Manning A, McElhinny C, Zakaria M. 2013. Contribution of illegal hunting, culling of pest species, road accidents and feral dogs to biodiversity loss in established oil-palm landscapes. Wildlife Research **40**:1. Available from <http://www.publish.csiro.au/?paper=WR12036>.
15. Barboza RRD, Lopes SF, Souto WMS, Fernandes-Ferreira H, Alves RRN. 2016. The role of game mammals as bushmeat In the Caatinga, northeast Brazil. Ecology and Society **21**:2. Available from <http://www.ecologyandsociety.org/vol21/iss2/art2/>.
16. Barnes RFW. 2002. The bushmeat boom and bust in West and Central Africa. Oryx **36**:95–120. Available from <http://www.journals.cambridge.org/abstract_S0030605302000443>.
17. Bitanyi S, Nesje M, Kusiluka LJ, Chenyambuga SW, Kaltenborn BP. 2012. Awareness and Perceptions of Local People about Wildlife Hunting in Western Serengeti Communities. Tropical Conservation Science **5**:208–224. Available from [https://doi.org/10.1177/194008291200500209](https://doi.org/10.1177%2F194008291200500209)
18. Bonwitt J, Kelly AH, Ansumana R, Agbla S, Sahr F, Saez AM, Borchert M, Kock R, Fichet-Calvet E. 2016. Rat-atouille: A Mixed Method Study to Characterize Rodent Hunting and Consumption in the Context of Lassa Fever. EcoHealth **13**:234–247. Springer US. Available from <http://link.springer.com/10.1007/s10393-016-1098-8>.
19. Borgerson C. 2015. The Effects of Illegal Hunting and Habitat on Two Sympatric Endangered Primates. International Journal of Primatology **36**:(1)74–93. Available from DOI: 10.1007/s10764-015-9812-x
20. Borgerson C. 2016. Optimizing conservation policy: the importance of seasonal variation in hunting and meat consumption on the Masoala Peninsula of Madagascar. Oryx **50**:405–418. Available from <https://doi.org/10.1017/S0030605315000307>
21. Borgerson C, McKean MA, Sutherland MR, Godfrey LR. 2016. Who hunts lemurs and why they hunt them. Biological Conservation **197**:124–130. Available from <http://dx.doi.org/10.1016/j.biocon.2016.02.012>.
22. Bragagnolo C, Correia R, Malhado ACM, de Marins M, Ladle RJ. 2017. Understanding non-compliance: Local people’s perceptions of natural resource exploitation inside two national parks in northeast Brazil. Journal for Nature Conservation **40**:64–76. Available from <http://dx.doi.org/10.1016/j.jnc.2017.09.006>.
23. Brooke AP, Tschapka M. 2002. Threats from overhunting to the flying fox, Pteropus tonganus, *(Chiroptera: Pteropodidae)* on Niue Island, South Pacific Ocean. Biological Conservation **103**:343–348. Available from <http://www.sciencedirect.com/science/article/pii/S0006320701001458>.
24. Brugiere D, Badjinca L, Silva C, Serra A. 2009. Distribution of Chimpanzees and Interactions with Humans in Guinea-Bissau and Western Guinea, West Africa. Folia Primatologica **80**:353–358. Available from <https://www.karger.com/Article/FullText/259335>.
25. Cano LS, Tellería JL. 2013. Local ecological knowledge as a tool for assessing the status of threatened vertebrates: a case study in Vietnam. Oryx **47**:177–183. Available from <http://www.journals.cambridge.org/abstract_S0030605311001669>.
26. Carpaneto GM, Fusari A. 2000. Subsistence hunting and bushmeat exploitation in central-western Tanzania. Biodiversity and Conservation **9**:1571–1585. Available from <https://doi.org/10.1023/A:1008943003752>.
27. Carvalho EAR, Pezzuti JCB. 2010. Hunting of jaguars and pumas in the Tapajós–Arapiuns Extractive Reserve, Brazilian Amazonia. Oryx **44**:610–612. Available from <http://www.journals.cambridge.org/abstract_S003060531000075X>.
28. Carvalho M, Palmeirim JM, Rego FC, Sole N, Santana A, Fa JE. 2015a. What motivates hunters to target exotic or endemic species on the island of São Tomé, Gulf of Guinea? Oryx **49**:278–286. Available from <http://www.journals.cambridge.org/abstract_S0030605313000550>.
29. Carvalho M, Rego F, Palmeirim JM, Fa JE. 2015b. Wild meat consumption on São Tomé Island, West Africa: implications for conservation and local livelihoods. Ecology and Society **20**:27. Available from <http://www.ecologyandsociety.org/vol20/iss3/art27/>.
30. Castilho LC, De Vlesschouwer KM, Milner-Gulland E, Chiavetti A. 2017. Hunting of mammal species in protected areas of the southern Bahian Atlantic Forest, Brazil. Oryx:1–11. Available from <https://doi.org/10.1017/S0030605317001247>
31. Castilho LC, Martinez RA, Giné GAF, Ribeiro GC, Schiavetti A. 2013. The thin-spined porcupine, *Chaetomys subspinosus (Rodentia: Erethizontidae),* within protected areas in the Atlantic Forest, Brazil: local knowledge and threats. Tropical Conservation Science **6**:796–810. Available from https://journals.sagepub.com/doi/pdf/10.1177/194008291300600607
32. Ceppi SL, Nielsen MR. 2014. A comparative study on bushmeat consumption patterns in ten tribes in Tanzania. Tropical Conservation Science **7**:272–287. Available from [https://doi.org/10.1177/194008291400700208](https://doi.org/10.1177%2F194008291400700208)
33. Chang CH, Barnes ML, Frye M, Zhang M, Quan R-C, Reisman LM, Levin SA, Wilcove DS. 2017. The pleasure of pursuit: recreational hunters in rural Southwest China exhibit low exit rates in response to declining catch. Ecology and Society **22**:43. Available from <http://www.ecologyandsociety.org/vol22/iss1/art43/>.
34. Cheok M, Mohid-Azlan J. 2018. Preliminary analysis on the hunting activities in selected areas in interior Sarawak. Malaysian Journal of Applied Biology **47**:37–43.
35. Chutia P, Solanki GS. 2013. Patterns of bird hunting in Arunachal Pradesh and implications for biodiversity conservation. Tropical Ecology **54**:263–267. Available from <http://tropecol.com/pdf/open/PDF_54_2/11-Chutia.pdf>
36. Coad L, Abernethy K, Balmford A, Manica A, Airey L, Milner-Gulland E. 2010. Distribution and Use of Income from Bushmeat in a Rural Village, Central Gabon. Conservation Biology **24**:1510–1518. Available from <http://doi.wiley.com/10.1111/j.1523-1739.2010.01525.x>.
37. Coad L, Schleicher J, Marthews TR, Starkey M. 2013. Social and Ecological Change over a Decade in a Village Hunting System, Central Gabon. Conservation Biology **27**:270–280. Availble from <https://doi.org/10.1111/cobi.12012>
38. Constantino P de AL. 2016. Deforestation and hunting effects on wildlife across Amazonian indigenous lands. Ecology and Society **21**:3. Available from <http://www.ecologyandsociety.org/vol21/iss2/art3/>.
39. Conteh A, Gavin MC. 2017. Influence of war on hunting patterns and pressure in Sierra Leone. Environmental Conservation **44**:131–138. Availble from <https://doi.org/10.1017/S0376892916000321>
40. Conteh A, Gavin MC, Solomon J. 2015. Quantifying illegal hunting: A novel application of the quantitative randomised response technique. Biological Conservation **189**:16–23. Available from <http://dx.doi.org/10.1016/j.biocon.2015.02.002>.
41. Cowlishaw G, Mendelson S, Rowcliffe Jm. 2005. Structure and Operation of a Bushmeat Commodity Chain in Southwestern Ghana. Conservation Biology **19**:139–149. Available from <https://doi.org/10.1111/j.1523-1739.2005.00170.x>
42. da Silva Neto BC, do Nascimento ALB, Schiel N, Nóbrega Alves RR, Souto A, Albuquerque UP. 2017. Assessment of the hunting of mammals using local ecological knowledge: an example from the Brazilian semiarid region. Environment, Development and Sustainability **19**:1795–1813. Available from <http://link.springer.com/10.1007/s10668-016-9827-2>
43. Dai C, Hu W. 2017. Hunting strategies employed by bird hunters with economic pursuit in the city of Guiyang, Southwest China. Journal for Nature Conservation **40**:33–41. Available from <http://dx.doi.org/10.1016/j.jnc.2017.09.005>.
44. de la Montaña E, Moreno-Sánchez R del P, Maldonado JH, Griffith DM. 2015. Predicting hunter behavior of indigenous communities in the Ecuadorian Amazon: insights from a household production model. Ecology and Society **20**:art30. Available from <http://www.ecologyandsociety.org/vol20/iss4/art30/>.
45. de Mattos Vieira MR, von Muhlen E, Shepard G. 2015. Participatory Monitoring and Management of Subsistence Hunting in the Piagaçu-Purus Reserve, Brazil. Conservation and Society **13**:254. Available from <http://www.conservationandsociety.org/text.asp?2015/13/3/254/170399>.
46. De Merode E, Cowlishaw G. 2006. Species Protection, the Changing Informal Economy, and the Politics of Access to the Bushmeat Trade in the Democratic Republic of Congo. Conservation Biology **20**:1262–1271. Available from <http://doi.wiley.com/10.1111/j.1523-1739.2006.00425.x>.
47. de Merode E, Homewood K, Cowlishaw G. 2004. The value of bushmeat and other wild foods to rural households living in extreme poverty in Democratic Republic of Congo. Biological Conservation **118**:573–581. Available from <http://linkinghub.elsevier.com/retrieve/pii/S0006320703004038>.
48. De Souza-Mazurek RR, Pedrinho T, Feliciano X, Hilário W, Gerôncio S, Marcelo E. 2000. Subsistence hunting among theWaimiri Atroari Indians in central Amazonia, Brazil. Biodiversity and Conservation **9**:579–596. Available from <http://link.springer.com/10.1023/A:1008999201747>.
49. Dounias E. 2016. From Subsistence to Commercial Hunting: Technical Shift in Cynegetic Practices Among Southern Cameroon Forest Dwellers During the 20th Century. Ecology and Society **21**:art23. Available from <http://www.ecologyandsociety.org/vol21/iss1/art23/>.
50. Duda R, Gallois S, Reyes-Garcia V. 2017. Hunting Techniques, Wildlife Offtake and Mar- Ket Integration. a Perspective From Individual Variations Among the Baka (Cameroon). African Study Monographs **38**:97–118. Available from https://doi.org/10.14989/225259
51. Dunn M, Estrada N, Smith DA. 2012. The coexistence of baird’s tapir *(Tapirus bairdii)* and indigenous hunters in northeastern Honduras. Integrative Zoology **7**:429–438. Availble from <https://doi.org/10.1111/j.1749-4877.2012.00322.x>
52. Eneji CVO, Ogar DA, Mubi AM, Husain MA. 2015. Gender Participation in Forest Resources Exploitation and Rural Development of the Forest Communities in Cross River State, Nigeria **1**:157–168. Available from <https://iiste.org/Journals/index.php/JNSR/article/viewFile/25943/26721>
53. Enuoh OOO, Bisong FE. 2014. Rural livelihoods vulnerabilities and commercial bushmeat hunting challenges in Cross River National Park, Nigeria. Natural Resources **5**:822–836. Available from DOI: [10.4236/nr.2014.513071](http://dx.doi.org/10.4236/nr.2014.513071)
54. Escamilla A. 2000. Habitat Mosaic, Wildlife Availability, and Hunting in the Tropical Forest of Calakmul, Mexico. Conservation Biology **14**:1592–1601. Available from <https://doi.org/10.1111/j.1523-1739.2000.99069.x>
55. Fa JE, Yuste JEG. 2001. Commercial bushmeat hunting in the Monte Mitra forests, Equatorial Guinea: extent and impact. Animal Biodiversity and Conservation **24**:31–52. Available from <http://abc.museucienciesjournals.cat/files/ABC-24-1-pp-31-52.pdf>
56. Franzen M. 2006. Evaluating the sustainability of hunting: a comparison of harvest profiles across three Huaorani communities. Environmental Conservation **33**:36. Available from <http://www.journals.cambridge.org/abstract_S0376892906002712>.
57. Franzen M, Eaves J. 2007. Effect of market access on sharing practices within two Huaorani communities. Ecological Economics **63**:776–785. Available from <http://linkinghub.elsevier.com/retrieve/pii/S0921800907000997>.
58. Fusari A, Carpaneto GM. 2006. Subsistence hunting and conservation issues in the game reserve of Gile, Mozambique. Biodiversity and Conservation **15**:2477–2495. Available from <http://link.springer.com/10.1007/s10531-004-8229-1>.
59. Gallina S, Pérez-Torres J, Guzmán-Aguirre CC. 2012. Use of the paca, Cuniculus paca (*Rodentia: Agoutidae*) in the Sierra de Tabasco State Park, Mexico. Revista de Biologia Tropical **60**:1345–1355. Available from <http://www.redalyc.org/articulo.oa?id=44923907032>
60. Gandiwa E. 2011. Preliminary assessment of illegal hunting by communities adjacent to the northern Gonarezhou National Park, Zimbabwe. Tropical Conservation Science **4**:445–467. Available from <http://journals.sagepub.com/doi/full/10.1177/194008291100400407>
61. Gandiwa E, Heitkönig IMA, Lokhorst AM, Prins HHT, Leeuwis C. 2013. Illegal hunting and law enforcement during a period of economic decline in Zimbabwe: A case study of northern Gonarezhou National Park and adjacent areas. Journal for Nature Conservation **21**:133–142. Available from <http://dx.doi.org/10.1016/j.jnc.2012.11.009>.
62. Gavin MC. 2007. Foraging in the fallows: Hunting patterns across a successional continuum in the Peruvian Amazon. Biological Conservation **134**:64–72. Available from <http://linkinghub.elsevier.com/retrieve/pii/S0006320706003181>.
63. Ghoddousi A, Soofi M, Kh. Hamidi A, Lumetsberger T, Egli L, Ashayeri S, Khorozyan I, H. Kiabi B, Waltert M. 2017. When pork is not on the menu: Assessing trophic competition between large carnivores and poachers. Biological Conservation **209**:223–229. Available from <https://linkinghub.elsevier.com/retrieve/pii/S0006320717303105>.
64. Gill DJC, Fa JE, Rowcliffe JM, Kümpel NF. 2012. Drivers of Change in Hunter Offtake and Hunting Strategies in Sendje, Equatorial Guinea. Conservation Biology **26**:1052–1060. Available from <http://doi.wiley.com/10.1111/j.1523-1739.2012.01876.x>.
65. Golden CD. 2009. Bushmeat hunting and use in the Makira Forest, north-eastern Madagascar: a conservation and livelihoods issue. Oryx **43**:386. Available from <http://www.journals.cambridge.org/abstract_S0030605309000131>.
66. Golden CD, Comaroff J. 2015. Effects of social change on wildlife consumption taboos in northeastern Madagascar. Ecology and Society **20**:41. Available from <http://www.ecologyandsociety.org/vol20/iss2/art41/>.
67. Goldman MJ, de Pinho JR, Perry J. 2013. Beyond ritual and economics: Maasai lion hunting and conservation politics. Oryx **47**:490–500. Available from <http://www.journals.cambridge.org/abstract_S0030605312000907>.
68. Gonedelé Bi S, Koné I, Béné JCK, Bitty EA, Yao KA, Kouassi BA, Gaubert P. 2017. Bushmeat hunting around a remnant coastal rainforest in Côte d’Ivoire. Oryx **51**:418–427. Available from <https://www.cambridge.org/core/product/identifier/S0030605315001453/type/journal_article>.
69. González-Marín RM, Moreno-Casasola P, Castro-Luna AA, Castillo A. 2017. Regaining the traditional use of wildlife in wetlands on the coastal plain of Veracruz, Mexico: ensuring food security in the face of global climate change. Regional Environmental Change **17**:1343–1354. Available from <http://link.springer.com/10.1007/s10113-016-0955-x>.
70. González JA. 2003. Harvesting, local trade, and conservation of parrots in the Northeastern Peruvian Amazon. Biological Conservation **114**:437–446. Available from <http://linkinghub.elsevier.com/retrieve/pii/S0006320703000715>.
71. Goodman SM. 2006. Hunting of Microchiroptera in south-western Madagascar. Oryx **40**:225. Available from <http://www.journals.cambridge.org/abstract_S0030605306000354>.
72. Grande-Vega M, Farfán MÁ, Ondo A, Fa JE. 2016. Decline in hunter offtake of blue duikers in Bioko Island, Equatorial Guinea. African Journal of Ecology **54**:49–58. Available from <http://doi.wiley.com/10.1111/aje.12260>.
73. Greengrass E. 2016. Commercial hunting to supply urban markets threatens mammalian biodiversity in Sapo National Park, Liberia. Oryx **50**:397–404. Available from <http://www.journals.cambridge.org/abstract_S0030605315000095>.
74. Grey-Ross R, Downs CT, Kirkman K. 2010. An Assessment of Illegal Hunting on Farmland in KwaZulu-Natal, South Africa: Implications for Oribi (*Ourebia ourebi*) Conservation. South African Journal of Wildlife Research **40**:43–52. Available from <http://www.bioone.org/doi/abs/10.3957/056.040.0104>.
75. Groff K, Axelrod M. 2013. A Baseline Analysis of Transboundary Poaching Incentives in Chiquibul National Park, Belize. Conservation and Society **11**:277. Available from <http://www.conservationandsociety.org/text.asp?2013/11/3/277/121031>.
76. Harrison M, Baker J, Twinamatsiko M, Milner-Gulland EJ. 2015. Profiling unauthorized natural resource users for better targeting of conservation interventions. Conservation Biology **29**:1636–1646. Available from <http://dx.doi.org/10.1111/cobi.12575>.
77. Harrison ME, Cheyne SM, Darma F, Ribowo DA, Limin SH, Struebig MJ. 2011. Hunting of flying foxes and perception of disease risk in Indonesian Borneo. Biological Conservation **144**:2441–2449. Available from <http://dx.doi.org/10.1016/j.biocon.2011.06.021>.
78. Hayward MW, Henschel P, O’Brien J, Hofmeyr M, Balme G, Kerley GIH. 2006. Prey preferences of the leopard (*Panthera pardus*). Journal of Zoology **270**:298–313. Available from <http://doi.wiley.com/10.1111/j.1469-7998.2006.00139.x>.
79. Holmern T, Mkama S, Muya J, Røskaft E. 2006. Intraspecific prey choice of bushmeat hunters outside the Serengeti National Park, Tanzania: a preliminary analysis. African Zoology **41**:81–87. Available from <http://www.bioone.org/doi/abs/10.3377/1562-7020%282006%2941%5B81%3AIPCOBH%5D2.0.CO%3B2>.
80. Ibbett H, Lay C, Phlai P, Song D, Hong C, Mahood SP, Milner-Gulland EJ. 2017. Conserving a globally threatened species in a semi-natural, agrarian landscape. Oryx:1–11. Available from <https://www.cambridge.org/core/product/identifier/S0030605316001708/type/journal_article>.
81. Ives IE, Platt SG, Tasirin JS, Hunowu I, Siwu S, Rainwater TR. 2008. Field Surveys, Natural History Observations, and Comments on the Exploitation and Conservation of *Indotestudo forstenii*, *Leucocephalon yuwonoi*, and *Cuora amboinensis* in Sulawesi, Indonesia. Chelonian Conservation and Biology **7**:240–248. Available from <http://www.bioone.org/doi/abs/10.2744/CCB-0718.1>.
82. Jacob D., Nelson I., Udoakpan U., Etuk U. 2015. Wildlife Poaching in Nigeria National Parks: A Case Study of Cross River National Park. International Journal of Molecular Ecology and Conservation **5**:1–7. Available from <http://biopublisher.ca/index.php/ijmec/article/view/1744>.
83. Jean Desbiez AL, Keuroghlian A, Piovezan U, Bodmer RE. 2011. Invasive species and bushmeat hunting contributing to wildlife conservation: the case of feral pigs in a Neotropical wetland. Oryx **45**:78–83. Available from <http://www.journals.cambridge.org/abstract_S0030605310001304>.
84. Jędrzejewski W, Carreño R, Sánchez-Mercado A, Schmidt K, Abarca M, Robinson HS, Boede EO, Hoogesteijn R, Viloria AL, Cerda H, Velásquez G, Zambrano-Martínezet S. 2017. Human-jaguar conflicts and the relative importance of retaliatory killing and hunting for jaguar *(Panthera onca)* populations in Venezuela. Biological Conservation **209**:524–532. Available from <https://linkinghub.elsevier.com/retrieve/pii/S0006320716307625>.
85. Jost Robinson CA, Daspit LL, Remis MJ. 2011. Multi-faceted approaches to understanding changes in wildlife and livelihoods in a protected area: a conservation case study from the Central African Republic. Environmental Conservation **38**:247–255. Available from <http://www.journals.cambridge.org/abstract_S0376892910000949>.
86. Kahler JS, Gore ML. 2012. Beyond the cooking pot and pocket book: Factors influencing noncompliance with wildlife poaching rules. International Journal of Comparative and Applied Criminal Justice **36**:103–120. Available from <http://www.tandfonline.com/doi/full/10.1080/01924036.2012.669913%5Cnhttp://www.tandfonline.com/doi/abs/10.1080/01924036.2012.669913>.
87. Kahler JS, Gore ML. 2015. Local perceptions of risk associated with poaching of wildlife implicated in human-wildlife conflicts in Namibia. Biological Conservation **189**:49–58. Elsevier Ltd. Available from <http://dx.doi.org/10.1016/j.biocon.2015.02.001>.
88. Kahler JS, Roloff GJ, Gore ML. 2012. Poaching Risks in Community-Based Natural Resource Management. Conservation Biology **27**:177–186. Available from <https://doi.org/10.1111/j.1523-1739.2012.01960.x>
89. Kamins AO, Restif O, Ntiamoa-Baidu Y, Suu-Ire R, Hayman DTS, Cunningham AA, Wood JLN, Rowcliffe JM. 2011. Uncovering the fruit bat bushmeat commodity chain and the true extent of fruit bat hunting in Ghana, West Africa. Biological Conservation **144**:3000–3008. Available from <http://dx.doi.org/10.1016/j.biocon.2011.09.003>.
90. Kaschula SA, Shackleton CM. 2009. Quantity and significance of wild meat off-take by a rural community in the Eastern Cape, South Africa. Environmental Conservation **36**:192. Available from <http://www.journals.cambridge.org/abstract_S0376892909990282>.
91. Katuwal HB, Parajuli K, Sharma S. 2016. Money Overweighed the Traditional Beliefs for Hunting of Chinese Pangolins in Nepal. Journal of Biodiversity & Endangered Species **04**:3–5. Available from <http://www.esciencecentral.org/journals/money-overweighed-the-traditional-beliefs-for-hunting-of-chinesepangolins-in-nepal-2332-2543-1000173.php?aid=83135>.
92. Kaul R, Jandrotia JS, McGowan PJK. 2004. Hunting of large mammals and pheasants in the Indian western Himalaya. Oryx **38**:426–431. Available from <http://www.journals.cambridge.org/abstract_S0030605304000808>.
93. Kiffner C, Peters L, Stroming A, Kioko J. 2015. Bushmeat consumption in the Tarangire-Manyara ecosystem, Tanzania. Tropical Conservation Science **8**:318–332. Available from [https://doi.org/10.1177/194008291500800204](https://doi.org/10.1177%2F194008291500800204)
94. Knapp EJ. 2007. Who Poaches? Household Economies of Illegal Hunters in Western Serengeti, Tanzania. Human Dimensions of Wildlife **12**:195–196. Available from <http://www.tandfonline.com/doi/abs/10.1080/10871200701323140>.
95. Knapp EJ. 2012. Why poaching pays: a summary of risks and benefits illegal hunters face in Western Serengeti, Tanzania. Tropical Conservation Science **5**:434–445. Available from [https://doi.org/10.1177/194008291200500403](https://doi.org/10.1177%2F194008291200500403)
96. Knapp EJ, Peace N, Bechtel L. 2017. Poachers and Poverty: Assessing Objective and Subjective Measures of Poverty among Illegal Hunters Outside Ruaha National Park, Tanzania. Conservation & Society **15**:24–32. Available from <http://www.conservationandsociety.org/text.asp?2017/15/1/24/201393>
97. Knapp EJ, Rentsch D, Schmitt J, Lewis C, Polasky S. 2010. A Tale of Three Villages: Choosing an Effective Method for Assessing Poaching Levels in Western Serengeti, Tanzania. Oryx **44**:178. Available from <https://doi.org/10.1017/S0030605309990895>
98. Kümpel NF, Milner-Gulland EJ, Cowlishaw G, Rowcliffe JM. 2009. Assessing Sustainability at Multiple Scales in a Rotational Bushmeat Hunting System. Conservation Biology **24**:861–871. Available from <http://doi.wiley.com/10.1111/j.1523-1739.2010.01505.x>.
99. Kümpel NF, Rowcliffe JM, Cowlishaw G, Milner-Gulland E. 2009. Trapper profiles and strategies: insights into sustainability from hunter behaviour. Animal Conservation **12**:531–539. Available from <https://doi.org/10.1111/j.1469-1795.2009.00279.x>
100. Leberatto AC. 2017. A Typology of Market Sellers of Protected Wildlife Across Peru. Deviant Behavior **38**:1352–1370. Available from <https://www.tandfonline.com/doi/full/10.1080/01639625.2016.1254963>.
101. LeBreton M, Prosser AT, Tamoufe U, Sateren W, Mpoudi-Ngole E, Diffo JLD, Burke DS, Wolfe ND. 2006. Patterns of bushmeat hunting and perceptions of disease risk among central African communities. Animal Conservation **9**:357–363. Available from <http://doi.wiley.com/10.1111/j.1469-1795.2006.00030.x>.
102. Li X, Jiang X. 2014. Implication of musk deer (Moschus spp.) depletion from hunter reports and dung transect data in northwest Yunnan, China. Journal for Nature Conservation **22**:474–478. Available from <http://dx.doi.org/10.1016/j.jnc.2014.05.004>.
103. Linder JM, Oates JF. 2011. Differential impact of bushmeat hunting on monkey species and implications for primate conservation in Korup National Park, Cameroon. Biological Conservation **144**:738–745. Available from <http://dx.doi.org/10.1016/j.biocon.2010.10.023>.
104. Lindsey PA, Romañach SS, Matema S, Matema C, Mupamhadzi I, Muvengwi J. 2011. Dynamics and underlying causes of illegal bushmeat trade in Zimbabwe. Oryx **45**:84–95. Available from <http://www.journals.cambridge.org/abstract_S0030605310001274>.
105. Liu F, McShea WJ, Garshelis DL, Zhu X, Wang D, Shao L. 2011. Human-wildlife conflicts influence attitudes but not necessarily behaviors: Factors driving the poaching of bears in China. Biological Conservation **144**:538–547. Available from <http://dx.doi.org/10.1016/j.biocon.2010.10.009>.
106. Loibooki M, Hofer H, Campbell KLI, East ML. 2002. Bushmeat hunting by communities adjacent to the Serengeti National Park, Tanzania: the importance of livestock ownership and alternative sources of protein and income. Environmental Conservation **29**:391–398. Available from <http://www.journals.cambridge.org/abstract_S0376892902000279>.
107. Luiselli L, Petrozzi F, Akani GC, Di Vittorio M, Amadi N, Ebere N, Dendi D, Amori G, Eniang EA. 2017. Rehashing bushmeat – Interview campaigns reveal some controversial issues about the bushmeat trade dynamics in Nigeria. Revue d’Ecologie (La Terre et la Vie) **72**:3–18. Available from <http://hdl.handle.net/2042/61887>
108. Luskin MS, Christina ED, Kelley LC, Potts MD. 2014. Modern Hunting Practices and Wild Meat Trade in the Oil Palm Plantation-Dominated Landscapes of Sumatra, Indonesia. Human Ecology 42:35–45. Available from DOI:10.1007/s 10745-013-9606-8
109. Luz A, Guèze M, Paneque-Gálvez J, Pino J, Macía M, Orta-Martínez M, Reyes-García V. 2015. How Does Cultural Change Affect Indigenous Peoples’ Hunting Activity? An Empirical Study Among the Tsimane’ in the Bolivian Amazon. Conservation and Society **13**:382. Available from <http://www.conservationandsociety.org/text.asp?2015/13/4/382/179879>.
110. Luz AC, Paneque-Gálvez J, Guèze M, Pino J, Macía MJ, Orta-Martínez M, Reyes-García V. 2017. Continuity and change in hunting behaviour among contemporary indigenous peoples. Biological Conservation **209**:17–26. Available from <https://linkinghub.elsevier.com/retrieve/pii/S0006320717301908>.
111. MacKenzie CA. 2018. Risk, Reciprocity and Retribution: Choosing to Extract Resources From a Protected Area. Ecological Economics **143**:314–323. Available from <http://dx.doi.org/10.1016/j.ecolecon.2017.10.009>
112. MacKenzie CA, Chapman CA, Sengupta R. 2012. Spatial patterns of illegal resource extraction in Kibale National Park, Uganda. Environmental Conservation **39**:38–50. Available from <http://www.journals.cambridge.org/abstract_S0376892911000282>.
113. Macmillan DC, Nguyen QA. 2013. Factors influencing the illegal harvest of wildlife by trapping and snaring among the Katu ethnic group in Vietnam. Oryx **48**:304–312. Available from [https://doi.org/10.1017/S0030605312001445](%20https://doi.org/10.1017/S0030605312001445)
114. Maisels F, Keming E, Kemei M, Toh C. 2001. Large mammal extinction and implications for montane forest conservation: a case study from the Kilum-Ijim Forest, North-West Province, Cameroon. Oryx **35**:322–331. Available from <https://doi.org/10.1046/j.1365-3008.2001.00204.x>
115. Martin A, Caro T, Kiffner C. 2013. Prey preferences of bushmeat hunters in an East African savannah ecosystem. European Journal of Wildlife Research **59**:137–145. Available from <http://link.springer.com/10.1007/s10344-012-0657-8>.
116. Martin A, Caro T, Mulder MB. 2012. Bushmeat consumption in western Tanzania: A comparative analysis from the same ecosystem. Tropical Conservation Science **5**:352–364. Available from [https://doi.org/10.1177/194008291200500309](https://doi.org/10.1177%2F194008291200500309)
117. Mendonça LET, Vasconcellos A, Souto CM, Oliveira TPR, Alves RRN. 2016. Bushmeat consumption and its implications for wildlife conservation in the semi-arid region of Brazil. Regional Environmental Change **16**:1649–1657. Available from <http://link.springer.com/10.1007/s10113-015-0901-3>.
118. Mgawe P, Mulder MB, Caro T, Martin A, Kiffner C. 2012. Factors affecting bushmeat consumption in the Katavi-Rukwa ecosystem of Tanzania. Tropical Conservation Science **5**:446–462. Available from <https://doi.org/10.1177/194008291200500404>
119. Mohsanin S, Barlow ACD, Greenwood CJ, Islam MA, Kabir MM, Rahman MM, Howlader A. 2013. Assessing the threat of human consumption of tiger prey in the Bangladesh Sundarbans. Animal Conservation **16**:69–76. Available from <http://doi.wiley.com/10.1111/j.1469-1795.2012.00571.x>.
120. Morcatty TQ, Valsecchi J. 2015. Social, biological, and environmental drivers of the hunting and trade of the endangered yellow-footed tortoise in the Amazon. Ecology and Society **20**:3. Available from <http://www.ecologyandsociety.org/vol20/iss3/art3/>.
121. Mwangi DK, Akinyi M, Maloba F, Ngotho M, Kagira J, Ndeereh D, Kivai S. 2016. Socioeconomic and Health Implications of Human—Wildlife Interactions in Nthongoni, Eastern Kenya. African Journal of Wildlife Research **46**:87–102. Available from <http://www.bioone.org/doi/10.3957/056.046.0087>.
122. Nash HC, Wong MHG, Turvey ST. 2016. Using local ecological knowledge to determine status and threats of the Critically Endangered Chinese pangolin (Manis pentadactyla) in Hainan, China. Biological Conservation **196**:189–195. Available from <http://dx.doi.org/10.1016/j.biocon.2016.02.025>.
123. Ndibalema VG, Songorwa AN. 2008. Illegal meat hunting in serengeti: dynamics in consumption and preferences. African Journal of Ecology **46**:311–319. Available from <http://doi.wiley.com/10.1111/j.1365-2028.2007.00836.x>.
124. Nielsen MR. 2006. Importance, cause and effect of bushmeat hunting in the Udzungwa Mountains, Tanzania: Implications for community based wildlife management. Biological Conservation **128**:509–516. Available from <http://linkinghub.elsevier.com/retrieve/pii/S0006320705004362>.
125. Nielsen MR, Jacobsen JB, Thorsen BJ. 2014. Factors determining the choice of hunting and trading bushmeat in the Kilombero Valley, Tanzania. Conservation Biology **28**:382–391. Available from <https://doi.org/10.1111/cobi.12197>.
126. Nielsen MR, Meilby H. 2013. Determinants of Compliance with Hunting Regulations Under Joint Forest Management in Tanzania. South African Journal of Wildlife Research **43**:120–137. Available from <http://www.bioone.org/doi/abs/10.3957/056.043.0210>.
127. Nielsen MR, Meilby H. 2015. Hunting and trading bushmeat in the Kilombero Valley, Tanzania: motivations, cost-benefit ratios and meat prices. Environmental Conservation **42**:61–72. Available from <http://www.journals.cambridge.org/abstract_S0376892914000198>.
128. Nielsen MR, Pouliot M, Meilby H, Smith-Hall C, Angelsen A. 2017. Global patterns and determinants of the economic importance of bushmeat. Biological Conservation **215**:277–287. Available from <http://dx.doi.org/10.1016/j.biocon.2017.08.036>.
129. Nielson MR, Meilby H, Smith-Hall C. 2016. How could the bushmeat trade in the Kilombero Valley of Tanzania be regulated? Insights from the rural value chain. Oryx **50**:84–93. Available from <https://doi.org/10.1017/S003060531400009X>
130. Nijman V, Oo H, Shwe NM. 2017. Assessing the Illegal Bear Trade in Myanmar Through Conversations With Poachers: Topology, Perceptions, and Trade Links to China. Human Dimensions of Wildlife **22**:172–182. Available from <https://doi.org/10.1080/10871209.2017.1263768>
131. Nuno A, Bunnefeld N, Naiman LC, Milner-Gulland EJ. 2013. A Novel Approach to Assessing the Prevalence and Drivers of Illegal Bushmeat Hunting in the Serengeti. Conservation Biology **27**:1355–1365. Available from DOI: 10.1017/S0030605317000862
132. Nuwer R, Bell D. 2014. Identifying and quantifying the threats to biodiversity in the U Minh peat swamp forests of the Mekong Delta, Vietnam. Oryx **48**:88–94. Available from <http://www.journals.cambridge.org/abstract_S0030605312000865>.
133. Nyaki A, Gray SA, Lepczyk CA, Skibins JC, Rentsch D. 2014. Local-Scale Dynamics and Local Drivers of Bushmeat Trade. Conservation Biology **28**:1403–1414. Available from <http://doi.wiley.com/10.1111/cobi.12316>.
134. O’Brien S, Emahalala ER, Beard V, Rakotondrainy RM, Reid A, Raharisoa V, Coulson T. 2003. Decline of the Madagascar radiated tortoise Geochelone radiata due to overexploitation. Oryx **37**:338–343. Available from <http://www.journals.cambridge.org/abstract_S0030605303000590>.
135. Obour BR, Asare R, Ankomah P, Larson T. 2016. Poaching and its Potential to Impact Wildlife Tourism: An Assessment of Poaching Trends in the Mole National Park in Ghana. Athens Journal of Tourism:169–192.
136. Ogunjemite BG, Ashimi TA. 2010. Hunting and Trading in the Nigerian Chimpanzee (Pan troglodytes vellerosus) in Gashaka-Mambilla region, Nigeria. Ethiopian Journal of Environmental Studies and Management **3**:62–69.
137. Ohl-Schacherer J, Shepard Jr. GH, Kaplan H, Peres CA, Levi T, Yu DW. 2007. The Sustainability of Subsistence Hunting by Matsigenka Native Communities in Manu National Park, Peru. Conservation Biology. Available from <https://www.jstor.org/stable/4620941>.
138. Oliva M, Montiel S, García A, Vidal L. 2014. Local Perceptions of Wildlife use in Los Petenes Biosphere Reserve, Mexico: Maya Subsistence Hunting in a Conservation Conflict Context. Tropical Conservation Science **7**:781–795. Available from [https://doi.org/10.1177/194008291400700414](https://doi.org/10.1177%2F194008291400700414)
139. Pangau-Adam M, Noske R, Muehlenberg M. 2012. Wildmeat or Bushmeat? Subsistence Hunting and Commercial Harvesting in Papua (West New Guinea), Indonesia. Human Ecology **40**:611–621. Available from <http://link.springer.com/10.1007/s10745-012-9492-5>.
140. Parathian HE, Maldonado AM. 2010. Human-nonhuman primate interactions amongst Tikuna people: perceptions and local initiatives for resource management in Amacayacu in the Colombian Amazon. American Journal of Primatology **72**:855–865. Available from <http://doi.wiley.com/10.1002/ajp.20816>.
141. Parry L, Barlow J, Peres CA. 2009. Allocation of hunting effort by Amazonian smallholders: Implications for conserving wildlife in mixed-use landscapes. Biological Conservation **142**:1777–1786. Available from <http://linkinghub.elsevier.com/retrieve/pii/S0006320709001530>.
142. Parry L, Barlow J, Peres CA. 2009. Hunting for Sustainability in Tropical Secondary Forests. Conservation Biology **23**:1270–1280. Available from <http://doi.wiley.com/10.1111/j.1523-1739.2009.01224.x>.
143. Pasachnik SA, Danoff-Burg JA, Antúnez EE, Corneil JP. 2014. Local knowlege and use of the Valle de Aguán Spiny-Tailed Iguana, *Ctenosaura melanosterna*, in Honduras. Herpetological Conservation and Biology **9**:436–447. Available from <http://www.herpconbio.org/Volume_9/Issue_2/Pasachnik_etal_2014.pdf>
144. Pattiselanno F, Lubis MI. 2014. Hunting at the Abun Regional Marine Protected Areas: A Link Between Wildmeat and Food Security. HAYATI Journal of Biosciences **21**:180–186. Available from <http://linkinghub.elsevier.com/retrieve/pii/S1978301916300936>.
145. Pereira PM, Valsecchi J, Queiroz H. 2017. Spatial patterns of primate hunting in riverine communities in Central Amazonia. Oryx:1–9. Available from <https://www.cambridge.org/core/product/identifier/S0030605317000199/type/journal_article>.
146. Radder L, Bech-Larsen T. 2008. Hunters’ Motivations and Values: A South African Perspective. Human Dimensions of Wildlife **13**:252–262. Available from <http://www.tandfonline.com/doi/abs/10.1080/10871200801986739>.
147. Randrianandrianina FH, Racey PA, Jenkins RKB. 2010. Hunting and consumption of mammals and birds by people in urban areas of western Madagascar. Oryx **44**:411–415. Available from <https://doi.org/10.1017/S003060531000044X>
148. Rao M, Htun S, Zaw T, Myint T. 2010. Hunting, Livelihoods and Declining Wildlife in the Hponkanrazi Wildlife Sanctuary, North Myanmar. Environmental Management **46**:143–153. Available from DOI:[10.1007/s00267-010-9519-x](https://doi.org/10.1007/s00267-010-9519-x)
149. Rao M, Myint T, Zaw T, Htun S. 2005. Hunting patterns in tropical forests adjoining the Hkakaborazi National Park, north Myanmar. Oryx **39**:292–300. Available from <http://www.journals.cambridge.org/abstract_S0030605305000724>.
150. Rao M, Zaw T, Htun S, Myint T. 2011. Hunting for a Living: Wildlife Trade, Rural Livelihoods and Declining Wildlife in the Hkakaborazi National Park, North Myanmar. Environmental Management **48**:158–167. Available from <http://link.springer.com/10.1007/s00267-011-9662-z>.
151. Remis MJ, Jost Robinson CA. 2012. Reductions in Primate Abundance and Diversity in a Multiuse Protected Area: Synergistic Impacts of Hunting and Logging in a Congo Basin Forest. American Journal of Primatology **74**:602–612. Available from <http://doi.wiley.com/10.1002/ajp.22012>.
152. Reuter KE, Randell H, Wills AR, Janvier TE, Belalahy TR, Sewall BJ. 2016. Capture, movement, trade, and consumption of mammals in Madagascar. PLoS ONE **11**:1–25. Available from <http://dx.doi.org/10.1371/journal.pone.0150305>.
153. Rist J, Milner-Gulland EJ, Cowlishaw G, Rowcliffe M. 2010. Hunter Reporting of Catch per Unit Effort as a Monitoring Tool in a Bushmeat-Harvesting System. Conservation Biology **24**:489–499. Available from <http://doi.wiley.com/10.1111/j.1523-1739.2010.01470.x>.
154. Rist J, Rowcliffe M, Cowlishaw G, Milner-gulland EJ. 2008. Evaluating measures of hunting effort in a bushmeat system. Biological Conservation **1**:2086–2099. DOI: 10.1016/j.biocon.2008.06.005.
155. Rodríguez M, Montiel S, Cervera MD, Castillo, MT, Naranjo EJ. 2012. The Practice and Perception of Batida (Group Hunting) In A Maya Community of Yucatan, Mexico. Journal of Ethnobiology **32**:212–227. Available from <http://www.bioone.org/doi/full/10.2993/0278-0771-32.2.212%0ABioOne>.
156. Rogan MS, Lindsey PA, Tambling CJ, Golabek KA, Chase MJ, Collins K, McNutt JW. 2017. Illegal bushmeat hunters compete with predators and threaten wild herbivore populations in a global tourism hotspot. Biological Conservation **210**:233–242. Available from <http://dx.doi.org/10.1016/j.biocon.2017.04.020>.
157. Rosa CA Da, Wallau MO, Pedrosa F. 2018. Hunting as the main technique used to control wild pigs in Brazil. Wildlife Society Bulletin **42**:111–118. Available from <http://doi.wiley.com/10.1002/wsb.851>.
158. Saif S, Tuihedur Rahman HM, MacMillan DC. 2018. Who is killing the tiger Panthera tigris and why? Oryx **52**:46–54. Available from <https://www.cambridge.org/core/product/identifier/S0030605316000491/type/journal_article>.
159. Sanei A, Zakaria M. 2011. Distribution pattern of the Persian leopard *(Panthera pardus saxicolor)* in Iran. Asia Life Sciences:**7**–18. Available from <https://www.researchgate.net/publication/258220250_Distribution_pattern_of_the_Persian_leopard_Panthera_pardus_saxicolor_in_Iran>
160. Scheffers BR, Corlett RT, Diesmos A, Laurance F. 2012. Local demand drives a bushmeat industry in a Philippine forest preserve. Tropical Conservation Science **5**:133–141. Available from [https://doi.org/10.1177/194008291200500203](https://doi.org/10.1177%2F194008291200500203)
161. Shaffer CA, Milstein MS, Yukuma C, Marawanaru E, Suse P. 2017. Sustainability and comanagement of subsistence hunting in an indigenous reserve in Guyana. Conservation Biology **31**:1119–1131. Available from <http://doi.wiley.com/10.1111/cobi.12891>.
162. Shaffer CA, Yukuma C, Marawanaru E, Suse P. 2018. Assessing the sustainability of Waiwai subsistence hunting in Guyana by comparison of static indices and spatially explicit, biodemographic models. Animal Conservation **21**:148–158. Available from <http://doi.wiley.com/10.1111/acv.12366>.
163. Siren AH, Wilkie DS. 2016. The effects of ammunition price on subsistence hunting in an Amazonian village. Oryx **50**:47–55. Available from <https://doi.org/10.1017/S003060531400026X>
164. Souza JB De. 2014. Hunting and use wildlife in an Atlantic Forest remnant of northeastern Brazil. Tropical Conservation Science **7**:145–160. Available from [https://doi.org/10.1177/194008291400700105](https://doi.org/10.1177%2F194008291400700105)
165. St John FA V, Mai C, Pei KJC. 2015. Evaluating deterrents of illegal behaviour in conservation: Carnivore killing in rural Taiwan. Biological Conservation **189**:86–94. Available from <http://dx.doi.org/10.1016/j.biocon.2014.08.019>.
166. Starr C, Nekaris KAI, Streicher U, Leung LKP. 2011. Field surveys of the Vulnerable pygmy slow loris Nycticebus pygmaeus using local knowledge in Mondulkiri Province, Cambodia. Oryx **45**:135–142. Available from <https://doi.org/10.1017/S0030605310001316>
167. Steinberg M. 2016. Jaguar conservation in southern Belize: Conflicts, perceptions, and prospects among mayan hunters. Conservation and Society **14**:13. Available from <http://www.conservationandsociety.org/text.asp?2016/14/1/13/182801>.
168. Steinmetz R, Srirattanaporn S, Mor-Tip J, Seuaturien N. 2014. Can community outreach alleviate poaching pressure and recover wildlife in South-East Asian protected areas? Journal of Applied Ecology **51**:1469–1478. Available from <https://doi.org/10.1111/1365-2664.12239>.
169. Stirnemann RL, Stirnemann IA, Abbot D, Biggs D, Heinsohn R. 2018. Interactive impacts of by-catch take and elite consumption of illegal wildlife. Biodiversity and Conservation **27**:931–946. Available from <https://doi.org/10.1007/s10531-017-1473-y>.
170. Struebig MJ, Harrison ME, Cheyne SM, Limin SH. 2007. Intensive hunting of large flying foxes Pteropus vampyrus natunae in Central Kalimantan, Indonesian Borneo. Oryx **41**:390–393. Available from <http://www.journals.cambridge.org/abstract_S0030605307002074>.
171. Sylvester O, Segura AG, Davidson-Hunt IJ. 2016. Wild Food Harvesting and Access by Household and Generation in the Talamanca Bribri Indigenous Territory, Costa Rica. Human Ecology **44**:449–461. Human Ecology. Available from <http://dx.doi.org/10.1007/s10745-016-9847-4>.
172. Teixeira PH, Thel T do, Ferreira JM, de Azevedo S, Junior WR, Lyra-Neves R. 2014. Local knowledge and exploitation of the avian fauna by a rural community in the semi-arid zone of northeastern Brazil. Journal of Ethnobiology and Ethnomedicine **10**:81. Available from <http://ethnobiomed.biomedcentral.com/articles/10.1186/1746-4269-10-81>.
173. Tieguhong JC, Zwolinski J. 2009. Supplies of bushmeat for livelihoods in logging towns in the Congo basin. Journal of Horticulture and Forestry **1**:65–80. Available from [http://www.academicjournals.org/JHF/abstracts/abstracts/abstract2009/Jul/Tieguhong and Zwolinski.htm](http://www.academicjournals.org/JHF/abstracts/abstracts/abstract2009/Jul/Tieguhong%20and%20Zwolinski.htm)
174. van Vliet N, Cruz D, Quiceno-Mesa MP, Jonhson Neves de Aquino L, Moreno J, Ribeiro R, Fa J. 2015. Ride, shoot, and call: wildlife use among contemporary urban hunters in Três Fronteiras, Brazilian Amazon. Ecology and Society **20**:8. Available from <http://www.ecologyandsociety.org/vol20/iss3/art8/>.
175. van Vliet N, Nasi R. 2008. Hunting for Livelihood in Northeast Gabon: Patterns, Evolution, and Sustainability. Ecology and Society **13**:33. Available from <https://www.ecologyandsociety.org/vol13/iss2/art33/>
176. Vasco C, Sirén A. 2016. Correlates of wildlife hunting in indigenous communities in the Pastaza province, Ecuadorian Amazonia. Animal Conservation **19**:422–429. Available from <http://doi.wiley.com/10.1111/acv.12259>.
177. Vega MG, Carpinetti B, Duarte J, Fa JE. 2013. Contrasts in Livelihoods and Protein Intake between Commercial and Subsistence Bushmeat Hunters in Two Villages on Bioko Island, Equatorial Guinea. Conservation Biology **27**:576–587. Available from <http://doi.wiley.com/10.1111/cobi.12067>.
178. Velho N, Laurance WF. 2013. Hunting practices of an Indo-Tibetan Buddhist tribe in Arunachal Pradesh, north-east India. Oryx **47**:389–392. Available from <http://www.journals.cambridge.org/abstract_S0030605313000252>.
179. Walters G, Schleicher J, Hymas O, Coad L. 2015. Evolving hunting practices in Gabon: lessons for community-based conservation interventions. Ecology and Society **20**:31. Available from http://dx.doi.org/10.5751/ES-08047-200431
180. Whytock RC, Morgan BJ, Awa T, Bekokon Z, Abwe EA, Buij R, Virani M, Vickery JA, Bunnefeld N. 2018. Quantifying the scale and socioeconomic drivers of bird hunting in Central African forest communities. Biological Conservation **218**:18–25. Available from <https://doi.org/10.1016/j.biocon.2017.11.034>.
181. Wilfred P, MacColl ADC. 2015. Local Perspectives on Factors Influencing the Extent of Wildlife Poaching for Bushmeat in a Game Reserve, Western Tanzania. International Journal of Conservation Science **6**:99–110.
182. Willcox AS, Nambu DM. 2007. Wildlife hunting practices and bushmeat dynamics of the Banyangi and Mbo people of Southwestern Cameroon. Biological Conservation **134**:251–261. Available from <http://linkinghub.elsevier.com/retrieve/pii/S0006320706003296>.
183. Wong A, Huaimei Y, Wong C, Abd.Shukor J. 2012. A study of hunting activity of Sambar deer and Bearded pig in Paitan Forest Reserve, Pitas, Sabah, Malaysia. Journal of Tropical Biology and Conservation **9**:35–47.
184. Yasuoka H, Hirai M, Kamgaing TOW, Kamdoum EC, Bobo KS. 2015. Changes in the composition of hunting catches in southeastern Cameroon: a promising approach for collaborative wildlife management between ecologists and local hunters. Ecology and Society **20**:25. Available from <http://www.ecologyandsociety.org/vol20/iss4/art25/>.
185. Zapata-Ríos G, Urgilés C, Suárez E. 2009. Mammal hunting by the Shuar of the Ecuadorian Amazon: is it sustainable? Oryx **43**:375. Available from <http://www.journals.cambridge.org/abstract_S0030605309001914>.

# Appendix S5 - Additional Analysis

**Table S5a**. Univariate binomial General Linear Model to explore relationship between whether journal has a policy on human research ethics (1) or not (0), and the type of research the journal publishes.

| Coefficients: | Est. | Std. Error | Z-value | Pr(z) |
| --- | --- | --- | --- | --- |
| (Intercept) | 1.012 | 0.584 | 1.733 | 0.083 |
| Ecology journal | -1.299 | 0.961 | -1.351 | 0.177 |
| Environmental journal | 17.555 | 3765.85 | 0.005 | 0.996 |
| Interdisciplinary journal | 17.555 | 3261.32 | 0.005 | 0.996 |

Signif. codes: 0 ‘***’ 0.001 ‘**’ 0.01 ‘*’ 0.05 ‘.’ 0.1 ‘ ’ 1.

**Table S5b.** Univariate binomial General Linear Model to explore relationship between whether journal has a policy on human research ethics (1) or not (0), and journal impact factor.

| Coefficients: | Est. | Std. Error | Z-value | Pr(z) | Odds |
| --- | --- | --- | --- | --- | --- |
| (Intercept) | -1.396 | 1.111 | -1.257 | 0.209 | 0.19 |
| Journal impact factor | 1.288 | 0.657 | 1.962 | 0.05* | 0.78 |

Signif. codes: 0 ‘***’ 0.001 ‘**’ 0.01 ‘*’ 0.05 ‘.’ 0.1 ‘ ’ 1
